# Invasive potential of cattle fever ticks in the southern United States

**DOI:** 10.1186/1756-3305-7-189

**Published:** 2014-04-17

**Authors:** John R Giles, A Townsend Peterson, Joseph D Busch, Pia U Olafson, Glen A Scoles, Ronald B Davey, J Mathews Pound, Diane M Kammlah, Kimberly H Lohmeyer, David M Wagner

**Affiliations:** 1 Center for Microbial Genetics and Genomics, Northern Arizona University, PO Box 4073, Flagstaff, AZ 86011, USA; 2 Biodiversity Institute, University of Kansas, Lawrence, KS 66045, USA; 3 USDA,ARS, Knipling-Bushland United States Livestock Insects Research Laboratory, 2700 Fredericksburg Rd Kerrville, TX 78028, USA; 4 USDA, ARS, Animal Diseases Research Unit, Washington State University, Pullman, WA 99164, USA; 5 USDA, ARS, Cattle Fever Tick Research Laboratory, Moore Air Base, Building 6419, 22675 N Moorefield Rd, Edinburg, TX 78541, USA

## Abstract

**Abstract':**

**Background**

For >100 years cattle production in the southern United States has been threatened by cattle fever. It is caused by an invasive parasite-vector complex that includes the protozoan hemoparasites *Babesia bovis* and *B. bigemina*, which are transmitted among domestic cattle via *Rhipicephalus* tick vectors of the subgenus *Boophilus*. In 1906 an eradication effort was started and by 1943 *Boophilus* ticks had been confined to a narrow tick eradication quarantine area (TEQA) along the Texas-Mexico border. However, a dramatic increase in tick infestations in areas outside the TEQA over the last decade suggests these tick vectors may be poised to re-invade the southern United States. We investigated historical and potential future distributions of climatic habitats of cattle fever ticks to assess the potential for a range expansion.

**Methods**

We built robust spatial predictions of habitat suitability for the vector species *Rhipicephalus (Boophilus) microplus* and *R. (B.) annulatus* across the southern United States for three time periods: 1906, present day (2012), and 2050. We used analysis of molecular variance (AMOVA) to identify persistent tick occurrences and analysis of bias in the climate proximate to these occurrences to identify key environmental parameters associated with the ecology of both species. We then used ecological niche modeling algorithms GARP and Maxent to construct models that related known occurrences of ticks in the TEQA during 2001–2011 with geospatial data layers that summarized important climate parameters at all three time periods.

**Results**

We identified persistent tick infestations and specific climate parameters that appear to be drivers of ecological niches of the two tick species. Spatial models projected onto climate data representative of climate in 1906 reproduced historical pre-eradication tick distributions. Present-day predictions, although constrained to areas near the TEQA, extrapolated well onto climate projections for 2050.

**Conclusions**

Our models indicate the potential for range expansion of climate suitable for survival of *R. microplus* and *R. annulatus* in the southern United States by mid-century, which increases the risk of reintroduction of these ticks and cattle tick fever into major cattle producing areas.

## Background

*Rhipicephalus* ticks and the pathogens they transmit present significant threats to cattle populations worldwide. The majority of the world’s estimated 1.2 billion cattle are at risk of exposure to disease-causing pathogens, which lead to significant losses from fatalities and decreased meat and milk production [1,2]. In particular, bovine babesiosis (cattle fever) has been a persistent challenge to domestic cattle production in the United States for over 100 years. Originally described by Smith & Kilborne [3], this disease system is driven by an efficient host-vector-parasite complex that includes the protozoan hemoparasites *Babesia bovis* and *B. bigemina*, which are transmitted by *Rhipicephalus (Boophilus) microplus* and *R. annulatus* among reservoir hosts (cattle). Babesiosis is nearly always fatal in naïve adult cattle; young calves may recover and remain asymptomatically infected throughout their adult life. Disease is difficult to detect in these chronically infected animals and they can serve as reservoirs for further transmission via competent tick vectors [4].

*Rhipicephalus microplus* (the southern cattle tick) and *R. annulatus* (the cattle tick) are successful ectoparasites of ungulates in North America; however, both are non-native to the region. The original range of *R. microplus* is tropical and sub-tropical forests of India, whereas *R. annulatus* is native to the Middle Eastern and Mediterranean regions. These species were among the first major agricultural pests introduced to the Americas by European colonists [5-7]. By the early 20^th^ century these tick species were broadly established, with *R. annulatus* ubiquitous in North and Central America and *R. microplus* in Central and South America [5,6]. They were responsible for widespread infestation and dispersal of bovine babesiosis, which severely impeded development of the cattle industry in the southern United States [8].

**Figure 1:**
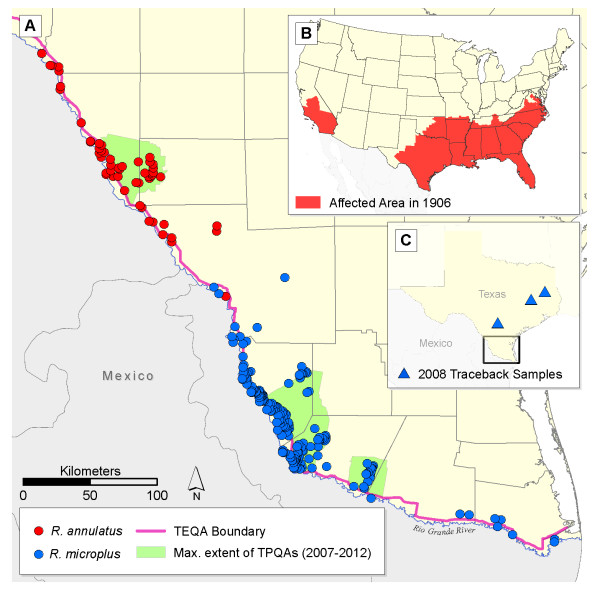
**Spatial distribution of*R. microplus*and*R. annulatus*samples utilized in this study. (A)** Distribution of both *R. microplus* and *R. annulatus* in the tick eradication quarantine area (TEQA) and the maximum extent of the temporary preventative quarantine areas (TPQAs) from 2007–2012. **(B)** historical distribution of cattle fever ticks before the CFTEP, and **(C)** location of our study area. The blue triangles in panel C mark three livestock feed lots where traceback ticks from Starr County were transported and later eradicated in April 2008.

In 1906, the United States Department of Agriculture (USDA) organized an eradication effort that effectively eliminated *R. annulatus* and *R. microplus* ticks and the *Babesia* parasites they transmit from the southern United States by 1943, except for a few locations in Florida and Texas. By 1960, cattle fever ticks and the pathogens they transmit were restricted to an area along the Texas-Mexico border. The tick eradication quarantine area (TEQA) is ~800 km long (covering an area >2,200 km^2^; Figure 1) and is rigorously monitored by the USDA-Animal and Plant Health Inspection Service, Veterinary Services (APHIS-VS). USDA-APHIS-VS employs horse mounted inspectors (sometimes known as “tick riders”) that patrol all areas within the TEQA for stray cattle and infestations of cattle fever ticks as part of the Cattle Fever Tick Eradication Program (CFTEP).

The risk of a re-invasion of cattle fever ticks beyond the TEQA remains a valid concern for the cattle industry. Historically, *R. microplus* and *R. annulatus* ticks were thought to be primarily ectoparasites of just cattle. However, recent research indicates that other wild ungulates, such as white-tailed deer (*Odocoileus virginianus*; hereafter, deer), can also serve as hosts of cattle fever ticks [9]. The use of these free-ranging hosts makes it more likely for cattle fever ticks to be transported beyond the TEQA in southern Texas [10]. Each year, the United States imports 1–2 million cattle from regions in Mexico where *R. microplus* and *R. annulatus* are endemic; some of these imported cattle may carry ticks resistant to standard control methods, such as acaricide dipping [11-14]. Since these cattle may also be infected with *Babesia*, all imported cattle must be transported beyond the TEQA to avoid the risk of endemic transmission in areas where *Rhipicephalus* ticks occur. Despite the formation of new temporary preventative quarantine areas (TPQAs, or blanket quarantine zones; Figure 1) in 2007, cattle fever tick infestations continued to increase both within the TEQA and TPQAs and beyond them in areas that were previously tick-free. In 2008, cases of *R. microplus* infestations in three feedlots were found in central and eastern Texas, up to 400 km northeast of the TEQA (Figure 1C). The alarming occurrence of cattle fever ticks far beyond the TEQA indicates that the threat of bovine babesiosis to cattle in the southern United States persists. In the event of a broad re-invasion, naive cattle would be highly susceptible to the disease; some estimates of mortality are as high as 90% [5,15]. Indeed, the consequences of re-establishment of cattle fever ticks would be costly—USDA estimates losses due to tick-vectored diseases could reach US $1 billion annually [16].

Control measures for tick-borne diseases have always focused on the tick vectors, and a thorough understanding of how the ticks interact with their environment is vital to continued efficacy of control measures [17-20]. Previous research efforts investigated population dynamics, parasite-host interactions, seasonal fluctuations, and physiological response to climate factors [19-27]. These studies added to a growing body of work that has elucidated many important variables in this complex ecological system. Attempts have been made to develop models to understand spatial dynamics of habitat suitability for cattle fever ticks, emphasizing ecological preferences and sensitivity to abiotic conditions [22-24,28]. Even with such progress, regional-scale high-resolution spatial models identifying environmental conditions contributing to the establishment and spread of this costly disease in cattle are lacking [24].

Global climate changes will certainly alter the spatial arrangement of suitable habitat for these important vectors [5,29,30]. Climate has emerged as a primary driver for distributions of both *R. microplus* and *R. annulatus*; a pattern common to many other vector-borne zoonotic disease systems [20,22,23,27,28,31-33]. The Intergovernmental Panel on Climate Change (IPCC) forecasts a 1-3°C increase in ambient surface temperature for the Gulf of Mexico region by mid-century see Additional file 1; [34]. As arthropod parasites, cattle fever ticks could respond to warming climates by expanding back into the southern United States [5,30]. As Sutherst [35] pointed out, each species responds differently in a changing environment, so an accurate understanding of climate change influences on this vector-disease system requires individual species-level approaches [36].

The purpose of this study was to identify climate parameters associated with persistence of *R. microplus* and *R. annulatus* and to develop high resolution spatial models that predict suitable environments for each species across the southern United States in past, present, and future climate scenarios. We focus on each species individually to identify areas at high risk of re-introduction facilitated by climate change. To the best of our knowledge, this study is the first to integrate tools from the fields of population genetics, spatial statistics, and ecological niche modeling to assess spatial and temporal trends in the cattle fever tick disease system.

## Methods

Spatial modeling of any biological phenomenon requires careful planning before analyses are performed. Often, data used in ecological niche models are not collected specifically for spatial predictions, and commonly used algorithms can be rendered null if fundamental assumptions are not met [36-42]. This study is no exception in terms of dedicated data collection; however, our methods aim to limit error introduced by distributional disequilibrium, sampling bias, and spatial autocorrelation. For the sake of brevity, many peripheral analyses and preparatory methods are relegated to appendices.

### Input data: occurrence data

We used a database of confirmed tick occurrences maintained by the joint CFTEP effort of the USDA-APHIS and the USDA-Agricultural Research Service (ARS), Cattle Fever Tick Research Laboratory in Edinburg, Texas. Thorough survey efforts by CFTEP mounted patrol inspectors from 1999–2011 provided a sample size of 314 and 63 infestations for *R. microplus* and *R. annulatus*, respectively (Figure 1; see Additional file 2 for a detailed list of occurrences). We sorted the occurrence data into two datasets: one “ALL”, which contains all occurrences in the original dataset, and the other “PERS” (persistent), which is intended to include tick collections from populations that may be persisting in the environment and is composed of occurrences ≤3 km from any infestation that had occurred ≥6 months before [see Additional file 3]. We chose 3 km as a distance threshold for defining persistence because our analysis of molecular variance (AMOVA) revealed little to no genetic differentiation from one year to the next among collections separated by ≤3 km. These genetic data suggest that at least some tick infestations are established ecologically, persisting long enough to be detected over multiple generations. An additional file offers more details on our use of genetic information see Additional file 4; [43].

Spatial distributions of both species are highly clustered within the TEQA, with many points occurring within the same 1 km^2^ raster cell of climate data used for spatial modeling. To avoid spurious results caused by spatial autocorrelation and pseudo-replication, we examined climate parameters relevant to *R. microplus* and *R. annulatus* individually (described below and in Additional file 5) via variogram analysis of spatial principal component layers that characterize multi-dimensional variation in the set of predictors selected for each species within areas near the TEQA [36]. Variography displays differences in raster layer values between pairs of sampled locations as a function of the distances separating them. An exponential linear model is then fitted to the variogram and important metrics, such as the nugget, range, and sill are calculated, which are then used to identify at what distance a variable is no longer correlated in space (spatial lag). We calculated spatial lag as the range value observed when the variogram model reaches 80% of the sill value. This method estimates the spatial lag as 7 km and 4.5 km for *R. microplus* and *R. annulatus*, respectively. Thus, we generated 10 replicate randomized subsets of both occurrence data sets such that each point is separated by ≥7 km for *R. microplus* and ≥4.5 km for *R. annulatus*. A more detailed account of this method can be found in additional material [see Additional file 6]. Persistent *R. annulatus* occurrences could not be included in our modeling exercises owing to small sample size [see Additional file 6: Table S1].

### Input data: environmental data

A variety of viewpoints have been expressed concerning relevance of climate in prediction of disease distributions [44-46]. However, when biological mechanisms that vlink vector distributions to climate variables are known, climate-based modeling becomes the best method for predicting disease distributions in the present and future [47,48]. As with many vector-borne disease systems, specific climate factors (e.g. ambient temperature, relative humidity, etc.) have a strong influence on the ecological success of ixodid ticks by altering vector generation time and survival rate [19,22,23,25-27,31,32,49,50]. Hence, our study focuses on identifying surrogate variables for defining suitable habitats statistically for both *R. microplus* and *R. annulatus*[51].

We developed geospatial data layers that summarize biologically relevant climate parameters across our study area for present, past, and future time scenarios. Data for present-day climate consisted of the 19 bioclimatic variables from the WorldClim database (spatial resolution: ~1 km; http://www.worldclim.org/) [52,53]. For climate parameters in the past, we obtained basic monthly temperature and precipitation products from the PRISM climate database (PRISM Climate Group, Oregon State University, http://prism.oregonstate.edu, created 4 Feb 2004) for the year 1906; the 19 bioclimatic variables were calculated via the ‘dismo’ package in R 2.15 [54]. Data layers representing future climate surfaces were obtained through the International Centre for Tropical Agriculture (CIAT) downscaled Global Climate Model (GCM) portal (http://www.ccafs-climate.org/): bioclimatic variables were derived from spatially disaggregated GCMs (four were chosen for this study: BCCR-BCM 2.0, CSIRO-Mk 3.5, MIROC 3.2-HIRES, NCAR-CCSM 3.0) under three future-carbon emissions scenarios (A1B, A2, B1) [34,55-59]. In all, we compiled 12 separate datasets that represent predicted climate parameters in 2050 at a spatial resolution of 1 km^2^.

Selection of predictor variables is a crucial decision that has significant impacts on spatial prediction [42,60]. Commonly, investigators choose specific biologically or ecologically relevant variables when building models for spatial prediction [36]. Although this method of variable selection is straightforward and intuitive, it can potentially introduce unwanted bias in model predictions [36,39,61,62]. As MacNally [63] aptly states, selection of independent predictor variables ought to be done using prior knowledge as well as theory.

We sought to identify a subset of the 19 bioclimatic variables that would serve as optimal predictors of the preferred habitats of both *R. microplus* and *R. annulatus* individually. Therefore, we performed an analysis of climate bias of the distributions of each tick species by comparing locations of known presence to locations of known absence within areas surrounding the TEQA; for detailed methods see Additional file 5. This quantitative approach to variable selection allowed us to extract six climate variables for each tick species that exhibit significant differences between presence and absence locations. We then explored possible interrelationships between climate parameters in each set of six variables via principal components analysis [36,64-67], because creation of uncorrelated orthogonal axes reduces potential effects of colinearity among predictor variables in modeling algorithms [67,68]. Hence, we included principal components 1 and 2 in our analysis, which describe >90% of the overall variation among the chosen climate variables.

### Model development

In the last decade diverse approaches have been explored in the ever-growing field of ecological niche modeling [69]. We explored two commonly used algorithms for spatial prediction, the Genetic Algorithm for Rule-Set Production (GARP) and a maximum entropy-based method (Maxent) [70-72]. GARP is a random-walk process that evolves a predictive rule (e.g. logistic regression, bioclimatic and range rules, etc.) with subsequent iterations until minimal improvement in the prediction of independent test data is achieved. Maxent forms model predictions by maximizing the entropy between the probability distribution of environmental variables at locations of presence to that of the user-selected study area.

Both algorithms use presence-only data coupled with automated, random sampling of pseudo-absences from a user-defined background area [37]. The use of presence-only data has evoked extensive discussion on the assumptions that are made when using modeling algorithms that create their own pseudo-absence data [41,69,70,73,74]. So, definition of the background landscape in presence-only modeling pursuits is of paramount importance, as shown by Barve *et al.*[37].

Elith *et al*. [70] point out that the background area (i.e. landscape of interest (*L*), as the referred document states) sampled for pseudo-absences is “suggested by the problem and defined by the ecologist”. The sampling scheme set in place by the USDA and APHIS made the definition of the background area a conveniently straightforward one. CFTEP mounted inspectors systematically patrol the TEQA in search of stray livestock infested with cattle fever ticks. So, locations within this thoroughly surveyed region where *R. microplus* or *R. annulatus* have not been observed would naturally serve as an appropriate pseudo-absence, were GARP or Maxent to sample one there. Therefore, we defined our background as the area of the eradication quarantine zone that is within 10 km of the US-Mexico border (roughly equal to the TEQA surveyed by USDA-APHIS inspectors).

**Figure 2:**
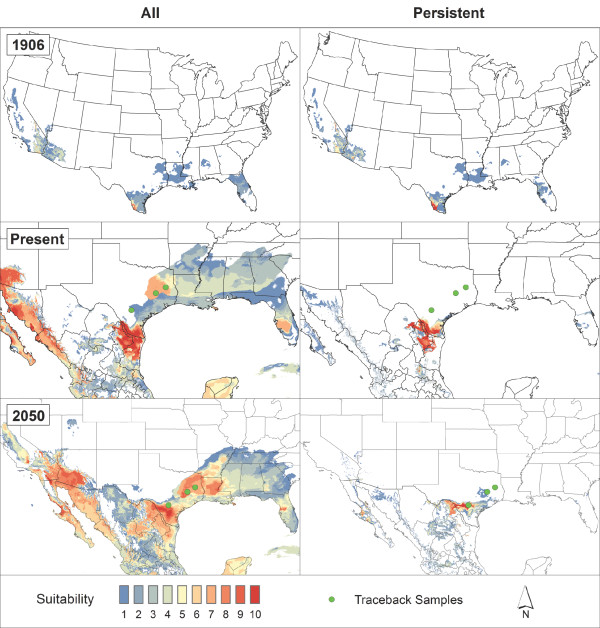
**Model predictions for*R. microplus.*** Models developed with ‘ALL’ and ‘PERS’ data used to predict climate suitability for *R. microplus* in 1906, present, and 2050 with three 2008 traceback samples shown as green filled circles.

**Figure 3:**
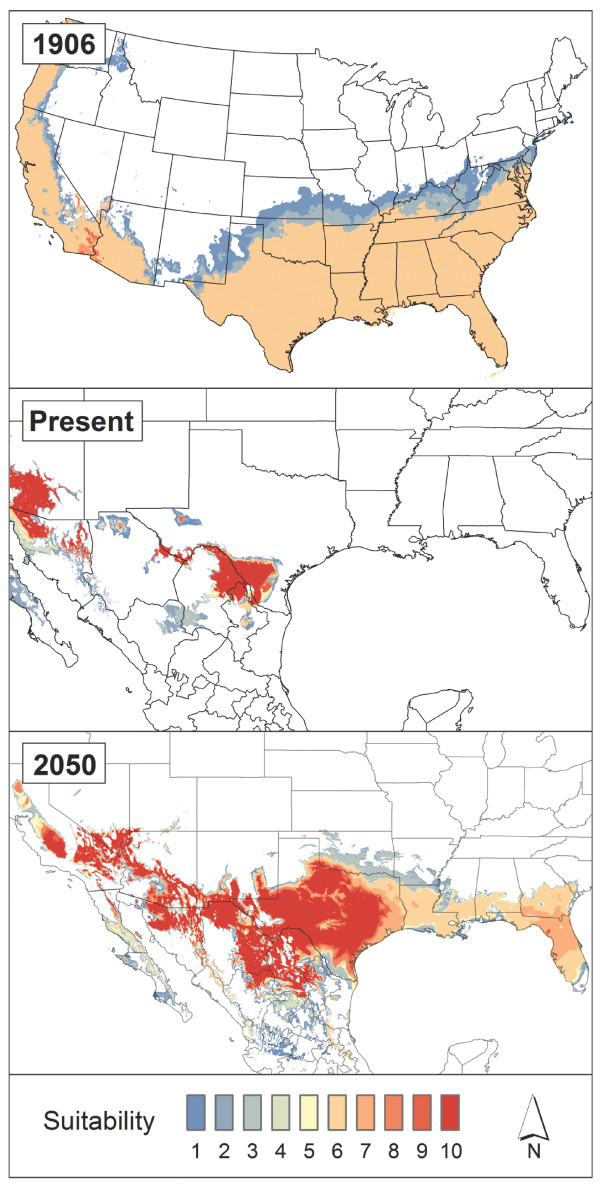
**Model predictions for*R. annulatus*.** Models developed with ‘ALL’ data identify areas of climate suitability for *R. annulatus* in 1906, present, and 2050.

A detailed account of algorithm parameters, model calibration and summary, and model evaluation is available in Additional file 7. Final model predictions are presented in terms of habitat suitability on a scale of 0–10, where 0 indicates that none of the random subset models agree on suitability, and 10 indicates that all models agree on suitability (Figures 2 and 3).

## Results

### Persistent occurrences

AMOVA of genotyped *R. microplus* samples from southern Texas indicated that southern cattle tick gene pools are highly admixed at distances ≤3 km, with stable gene pools (*F*_ST_ < 0.05) from one generation to the next (3–6 months) [see Additional file 4]. The temporally-based AMOVA technique was initially employed to identify locations where cattle fever ticks may be ecologically established, against a background of occurrences created by human-aided dispersal events. In light of its success with our data, we recommend this method as an effective tool to assess persistence locations for diverse spatial modeling studies in other species.

When a subsample of “persistent” occurrences was taken with the spatial and temporal constraints inferred from the AMOVA, we identified a climatic signature distinct from that derived from all points. Specifically, six climate variables unique to persistent locations for both *R. microplus* and *R. annulatus* had distributions significantly different from non-persistent ones; these variables summarize environmental conditions related to interactions between temperature extremes and moisture [see Additional file 5]. Coincidentally, the joint role of temperature and ambient humidity has been noted repeatedly as an important determinant of population dynamics of cattle fever ticks. Particularly, desiccation in larval stages appears to be a strong driver of success from one generation to the next [20,31]. For *R. microplus*, key climate variables were annual mean temperature (Bio 1), minimum temperature of coldest month (Bio 6), mean temperature of wettest quarter (Bio 8), mean temperature of driest quarter (Bio 9), mean temperature of coldest quarter (Bio 11), and precipitation seasonality (Bio 15). For *R. annulatus*, climate variables selected were annual mean temperature (Bio 1), mean diurnal temperature range (Bio 2), temperature seasonality (Bio 4), maximum temperature of warmest month (Bio 5), mean temperature of coldest quarter (Bio 11), and precipitation of wettest quarter (Bio 16).

Within the TEQA, distributions of the two cattle fever tick species are segregated, with *R. microplus* found to the southeast of Laredo, Texas, and *R. annulatus* to the northeast [75]. Our climate bias analyses indicated that the two species are also distinct with regards to climate parameters (see Additional file 5, including temperature seasonality (Bio 4), minimum temperature of coldest month (Bio 6), temperature annual range (Bio 7), mean temperature of driest quarter (Bio 9), mean temperature of coldest quarter (Bio 11), and precipitation of wettest month (Bio 13). Differences in climate preferences between *R. microplus* (in general – hot and humid) and *R. annulatus* (drier and cooler) may explain why the two species separate into distinct northern and southern distributions within the quarantine zone (Figure 1). Also it suggests that *R. annulatus*, in view of its greater tolerance for seasonal extremes and temperature and precipitation minima, is the species most likely to re-establish in the greater United States.

### Model predictions

Based on independent regional subsets of occurrence points excluded from model calibration, Maxent out-performed GARP with higher partial-AUC scores; therefore, only its results are presented here [see Additional file 7: Table S1]. Figure 2 displays historical (1906) spatial predictions for *R. microplus*, wherein models built with both ALL and PERS occurrences identified suitable areas across southern Texas, Louisiana, and Florida, along with southern California and parts of Arizona. Cattle fever ticks were previously known throughout the southern United States and parts of the Midwest; however, *R. microplus* is thought to have been responsible for infestations in the more humid southern regions, since it was originally from the Tropics [22]. Although these models recreate only a portion of the historical range for *R. microplus*, it is promising that they effectively extrapolate climate patterns found in the TEQA into environments that previously supported populations of this species. Figure 2 also shows present-day models for *R. microplus*, where ALL and PERS predictions show high suitability near the TEQA. However, the ALL model identified suitable areas across the southern United States and Florida; projecting these same models onto future (2050) climate scenarios yielded a similar spatial pattern, but with areas of highest suitability shifted north and east.

The three cases of *R. microplus* that were found in central and eastern Texas in 2008 occurred in areas that are already predicted as moderately suitable by the ALL present day model. Further, both ALL and PERS models anticipated increased suitability at these same locations by 2050 (Figure 2). The infested cattle in this instance were intercepted within a few days of transport, preventing ecological establishment of the ticks that they carried. However, if they had not been identified in a timely manner, these ticks could have established populations in these suitable areas; according to our projections, this scenario becomes even more likely under future climate conditions.

Models for *R. annulatus* (Figure 3) were built with ALL occurrence data because the PERS classification of occurrences returned too small a sample size for spatial prediction [see Additional file 6: Table S1]. When the *R. annulatus* model was projected onto 1906 climate data, the resulting distribution closely resembles the outline of counties that historically reported infestations of cattle fever ticks (Figures 1B and 3). This is perhaps not surprising because *R. annulatus* is thought to have been responsible for most cattle fever tick infestations during the height of the cattle fever era, possibly because it is more tolerant of dryer and colder conditions than *R. microplus*.

Model predictions for the present day conservatively predicted highly suitable habitat for *R. annulatus* in southern Texas and Arizona (Figure 3). When the same model was projected onto future climate data (2050), favourable environments demonstrated a sizeable expansion in area, across all of Texas and the southern United States, including Florida. Even a small increase (2-3°C) in annual mean temperature as projected by the IPCC greatly induces increased suitability for *R. annulatus*, far beyond the TEQA and TPQAs [see Additional file 1].

## Discussion

This paper presents the first large-scale and high-resolution spatial models of suitability for cattle fever ticks in the United States across multiple time scenarios, and our results are congruent with the historical ranges of both *R. microplus* and *R. annulatus* in the US. Bram *et al*. [5] noted that *R. microplus* infestations on deer were responsible for the prolonged persistence of cattle fever in Florida during the height of the CFTEP. Coincidentally, our *R. microplus* models for 1906 show suitability in Florida, which suggests the persistence of tick populations despite CFTEP efforts until the 1940’s may have been aided by suitable climatic conditions in that region (Figure 2). *R. annulatus* is considered the main tick species responsible for the full extent of cattle fever occurrence across the southern United States [8]. Our models agree with this notion, as they predict environmental suitability across broad areas of the south-eastern United States and southern California, with range limits that are similar to the counties reporting cattle fever ticks in 1906 (Figures 1B and 3). The thorough efforts of USDA-APHIS and the Texas Animal Health Commission currently restrict occurrences of cattle fever ticks to southern Texas. Our models for the present day indicate high suitability in areas surrounding this zone, which suggests that suitable tick habitat is limited climatically to areas near the Rio Grande. However, this result likely stems from spatially conservative predictions produced by the nature of our occurrences used in our modeling efforts, which were all clustered within the TEQA.

Based on our models, we anticipate potential for a dramatic range shift to the north and east of the TEQA for both *R. microplus* and *R. annulatus* by midcentury. A recent study by Pérez de León *et al.*[76] used wavelet analysis to identify a 30-yr cyclical pattern in historical records (1959–2011) of cattle fever tick infestations in southern Texas—a pattern potentially driven by broad-scale climate phenomena, such as the El Niño Southern Oscillation (ENSO) and the Accumulated Cyclone Energy Index (ACE). Accordingly, the current increase in cattle fever tick infestations along the quarantine zone is part of a recurring cycle that may be currently decreasing, with another upsurge in tick occurrence expected around 2050, potentially in areas that our spatial models predict as suitable far beyond the current TEQA.

Model projections onto likely 2050 conditions predict increases in suitable areas for each species north and east of the TEQA (Figures 2 and 3). The particular direction of the north-eastern range shift observed in this study is congruent with broad expectations that dynamic distribution changes will occur in vector-borne diseases in temperate regions [44,45,76]. When compared with the updated climate types presented in Peel *et al.*[77], future models exhibit movement from arid/desert steppe climate towards, what is currently classified as, a seasonal temperate region that extends through the southern United States. In a parallel vector-based disease system with *Theileria* (the causative agent of theileriosis, or East Coast Fever), Olwoch *et al.*[30] noted increases in prevalence of the tick vector *Rhipicephalus appendiculatus* upon elevated minimum temperatures in sub-Saharan Africa, and reduced prevalence with increased temperatures in already-hot and/or arid regions [78,79]. Increases in temperature minima over the course of the season can contribute to disease incidence by reducing pathogen incubation period, expediting vector generation time, larval survival rate, and overall population growth rate [48,80]. Beyond temperature extremes, changes in seasonal precipitation regimes impact tick life cycles via changes in vegetation-based micro-climate that provide stable seasonal and diurnal humidity at egg-laying and larval development sites, in addition to host questing opportunities [78,79]. Consequently, changes in macro and micro-climatic extremes as a result of climate changes can directly impact range expansions and range shifts of tick-borne disease systems [78,81]. Based on their individual climatic tolerances, this suggests alternative outcomes for *R. microplus* and *R. annulatus* in temperate regions of the southern United States as a possible consequence of climate change [28,30,44,45,80,82].

An increase in total area of suitability in the future does not guarantee the presence of the species in those new areas [44,47,76,83,84]. The models we developed here offer several unique insights into the natural history, ecology, and potential distribution of both *R. microplus* and *R. annulatus*. However, several caveats must accompany interpretation of our models. First, models are only as good as the input data [81]. Our spatial predictions originate from occurrence data from the TEQA only. Extrapolating such models across novel environments can be perilous because these models were calibrated with a restricted set of environments relative to the complete range of tolerance of the species (i.e. across a considerably smaller area compared to its natively accessible range). We regard the marginal set of occurrences used for model training as the foremost contributor to our conservative spatial predictions. Hence, we made a thorough effort to remove bias in the data and adjusted algorithm parameters to allow for extrapolation outside the initial range of training values. Second, our chosen predictor variables characterize habitat as suitable based on climate only. The biogeography of disease systems is complex, and requires appropriate land cover, as well as factors related to ungulate hosts for transmission to occur.

In addition to potential climate change, other factors also present potential challenges for future control of the cattle fever tick and *Babesia* disease system. The disease system currently exists in an ecologically imbalanced state as a result of habitat fragmentation, urbanization, land-use changes, and human-imposed species disequilibria, making it especially susceptible to the uncertain effects of global change [76]. White-tailed deer are known hosts for ixodid ticks, and were recently found to be sero-positive for exposure to *Babesia* spp. in Texas and northern Mexico [9,85,86]. Since formation of the CFTEP, the population size of this free-ranging host has increased dramatically in Texas (from ~10,000 to ~3-4 million), which significantly improves the dispersal capabilities of both *R. microplus* and *R. annulatus*[5,9,87]. Additionally, some *Rhipicephalus* tick populations in Mexico have now evolved resistance to organophosphates and other acaricides owing to liberal use in control efforts, which now confound future use of chemicals in the CFTEP [13,14,88].

The tick infestations in east-central Texas in 2008 illustrate the substantial risk of the re-invasion of cattle fever ticks. Although these instances of quarantine breach were noted promptly, they transpired in areas suitable for persistent populations. Given the conservative nature of our spatial predictions and the historical distribution of cattle fever ticks, introductions could also potentially occur further from the TEQA. If cattle fever ticks were to reach their former distribution, large deer populations, acaricide resistance, and increased habitat suitability would pose considerable challenges to a re-eradication effort [5]. These developments indicate that other changes, in addition to climate change, may modify cattle fever tick distributions in the southern United States [46,47].

## Conclusions

Many factors may permit the prevalence of a disease to increase over time. The biology and ecology of the host-vector-pathogen system is complex, even without human intervention. Based on our model results, we predict a dramatic range shift and increase of suitable climate for *R. microplus* and *R. annulatus* into temperate regions in the southern United States by midcentury. The risk imposed by global change and the movement and/or control of species integral to this system presents unique future challenges that emphasize the increasing risk of a re-invasion of cattle fever ticks. Should the CFTEP be compromised, climate-based spatial predictions of ecological suitability for cattle fever ticks may be the best predictor of cattle fever tick prevalence in a changing world.

The patterns discussed here are important not only for the ongoing management of the cattle fever system. They are also broadly applicable to global research conducted on a vast array of zoonotic diseases, which often manifest from a symphony of multi-dimensional variables resulting in disease occurrence, absence, and persistence. Studies like the one presented here fill an integral role in inter-disciplinary research that attempts to triangulate central processes driving disease emergence and occurrence which are vital for a comprehensive understanding of the dynamics of infectious ecological diseases.

## Competing interests

The authors report no competing interests.

## Authors’ contributions

JRG, ATP, DMW conceived the studies. PUO, GAS, RBD, JMP, DMK, KHL provided data and other information critical to the study. JRG carried out most analyses and wrote the initial manuscript. JDB performed the genotyping and AMOVA analysis. All authors contributed to the development of the final manuscript and approved its final version.

## Supplementary Material

Additional file 1 **Predicted temperature increases in the southern United States.** This figure displays annual mean temperature values that are within the observed range of climate values at sites of cattle fever tick presence used in this study (green pixels). Yellow, orange, and red pixels represent where this temperature range would be observed under the predicted IPCC temperature increases of 1, 2, and 3°C respectively. Click here for file

Additional file 2 Comprehensive table of all tick occurrences employed in this study for model calibration. Click here for file

Additional file 3 **An example of spatial selection of persistent occurrences in *Rhipicephalus microplus.***
Click here for file

Additional file 4 Analysis of Molecular Variance (AMOVA). Click here for file

Additional file 5 Climate bias analysis. Click here for file

Additional file 6 Spatial autocorrelation & data rarefaction. Click here for file

Additional file 7 Algorithm parameters and model summarization. Click here for file
