# Supplementary material for: Invasive potential of cattle fever ticks in the southern United States
Source: Parasit Vectors. 2014 Apr 17;7:189. doi: 10.1186/1756-3305-7-189 (PMC4021724; doi:10.1186/1756-3305-7-189)

Additional file 1 – Predicted temperature increases in the southern United States

This figure displays annual mean temperature values that are within the observed range of climate values at sites of cattle fever tick presence used in this study (green pixels). Yellow, orange, and red pixels represent where this temperature range would be observed under the predicted IPCC temperature increases of 1, 2, and 3°C respectively.

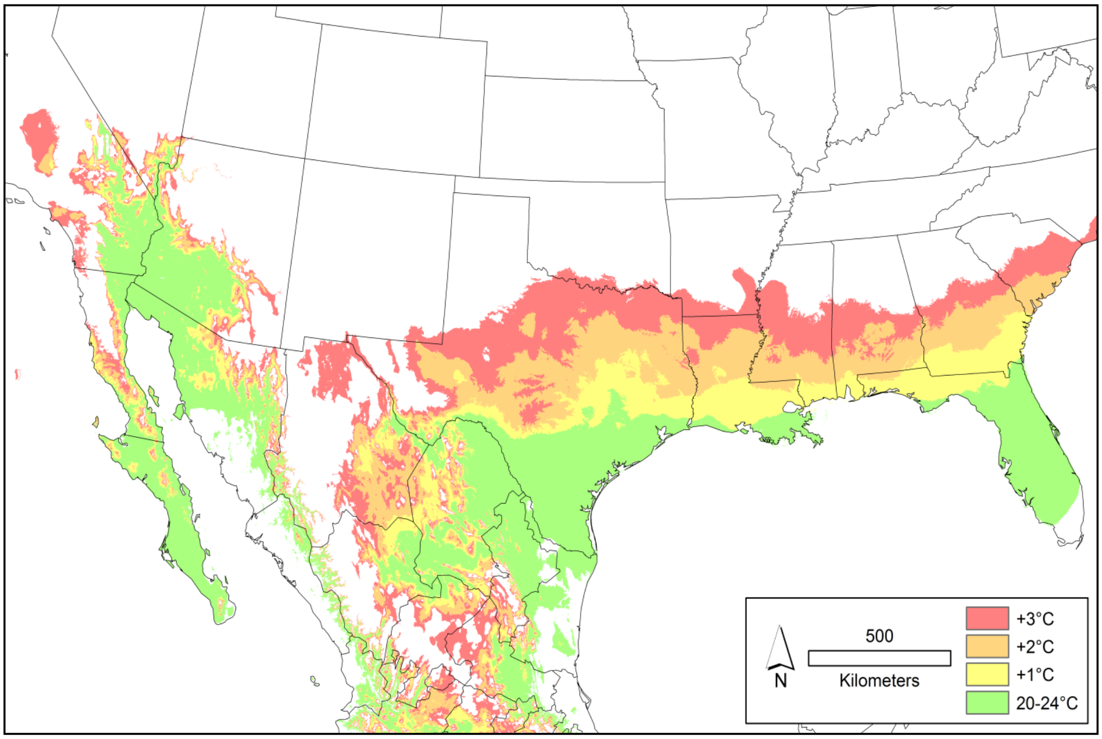

Supplement: Additional file 1 — Predicted temperature increases in the southern United States. This figure displays annual mean temperature values that are within the observed range of climate values at sites of cattle fever tick presence used in this study (green pixels). Yellow, orange, and red pixels represent where this temperature range would be observed under the predicted IPCC temperature increases of 1, 2, and 3°C respectively. [file 1756-3305-7-189-S1.pdf]
