# Supplementary material for: Invasive potential of cattle fever ticks in the southern United States
Source: Parasit Vectors. 2014 Apr 17;7:189. doi: 10.1186/1756-3305-7-189 (PMC4021724; doi:10.1186/1756-3305-7-189)
Supplement: Additional file 2 — Comprehensive table of all tick occurrences employed in this study for model calibration. [file 1756-3305-7-189-S2.pdf]

Additional file 2 – Comprehensive table of all tick occurrences employed in this study for model calibration

Species	Host Animal	Date	Year	County
R. annulatus	Native Cattle	Jul 12	2007	Maverick
R. annulatus	Native Cattle	Aug 25	2003	Webb
R. annulatus	Native Cattle	Jun 29, Jul 1	2005	Webb
R. annulatus	Native Cattle	May 6	2008	Webb
R. annulatus	Native Cattle	Jun 4	2009	Webb
R. annulatus	Native Cattle	Jun 4	2009	Webb
R. annulatus	Native Cattle	Nov 29	2007	Webb
R. annulatus	Native Cattle	Nov 1	2007	Webb
R. annulatus	Native Cattle	Mar 7	2008	Webb
R. annulatus	Native Cattle	Jul 7	2006	Webb
R. annulatus	Native Cattle	Nov 12	2004	Webb
R. annulatus	Native Cattle	Feb 14	2005	Webb
R. annulatus	White-tailed Deer	Jan 23	2007	Maverick
R. annulatus	Native Cattle	Sep 5	2007	Maverick
R. annulatus	Native Cattle	Mar 14	2008	Maverick
R. annulatus	Native Cattle	Apr 18	2008	Dimmit
R. annulatus	Exotic Fallow Deer	Nov 15	2007	Dimmit
R. annulatus	Native Cattle	Jan 15	2009	Dimmit
R. annulatus	Native Cattle	Jan 15	2009	Dimmit
R. annulatus	Native Cattle	Nov 13	2007	Dimmit
R. annulatus	Native Cattle	May 12	2006	Maverick
R. annulatus	Exotic Red Deer	Aug 20	2008	Maverick
R. annulatus	Native Cattle	Dec 16	2008	Dimmit
R. annulatus	Native Cattle	Sep 3	2008	Maverick
R. annulatus	Native Cattle	Aug 7	2008	Dimmit
R. annulatus	Native Cattle	Feb 11	2009	Dimmit
R. annulatus	Native Cattle	Aug 17	2007	Dimmit
R. annulatus	Horses	Feb 26	2010	Maverick
R. annulatus	Native Cattle	Aug 7	2007	Dimmit
R. annulatus	Native Cattle	Dec 23	2008	Maverick
R. annulatus	Native Cattle	May 31	2007	Maverick
R. annulatus	Native Cattle	May 14	2009	Dimmit
R. annulatus	Native Cattle	May 14	2009	Dimmit
R. annulatus	Native Cattle	Sep 18	2008	Maverick
R. annulatus	Native Cattle	Sep 26	2008	Maverick

R. annulatus	Native Cattle	Dec 19	2008	Maverick
R. annulatus	Native Cattle	Nov 6	2007	Dimmit
R. annulatus	Native Cattle	Sep 27	2007	Dimmit
R. annulatus	Native Cattle	Jul 24	2007	Maverick
R. annulatus	Native Cattle	Oct 22	2009	Maverick
R. annulatus	Native Cattle	Oct 22	2009	Maverick
R. annulatus	Native Cattle	Sep 27	2007	Dimmit
R. annulatus	Native Cattle	Dec 7	2006	Maverick
R. annulatus	Native Cattle	Aug 1	2007	Maverick
R. annulatus	Native Cattle	Dec 07	2006	Maverick
R. annulatus	Exotic Red Deer	Oct 26	2007	Maverick
R. annulatus	Mexico Cattle	Mar 23	2007	Maverick
R. annulatus	Native Cattle	Jun 15	2007	Maverick
R. annulatus	Native Cattle	Feb 14	2005	Maverick
R. annulatus	Native Cattle	Oct 22	2009	Maverick
R. annulatus	Exotic Red Deer	Dec 18	2007	Maverick
R. annulatus	Exotic Red Deer	Jun 16, Oct 30	2009	Maverick
R. annulatus	Native Cattle	Oct 4	2007	Maverick
R. annulatus	Mexico Cattle	Mar 22	2000	Maverick
R. annulatus	Native Cattle	Oct 22	2009	Maverick
R. annulatus	Native Cattle	Oct 10	2006	Kinney
R. annulatus	Native Cattle	Jul 28, Aug 13, Sep 10	2009	Kinney
R. annulatus	Native Cattle	Jul 28, Aug 13, Sep 10	2009	Kinney
R. annulatus	Native Cattle	Mar 9	2005	Kinney
R. annulatus	Native Cattle	Feb 10	2009	Kinney
R. annulatus	Native Cattle	Feb 10	2009	Kinney
R. microplus	Native Cattle	Nov 15	2004	Cameron
R. microplus	Native Cattle	Jun 14	2007	Cameron
R. microplus	Nilgai Antelope	Mar 10	2009	Cameron
R. microplus	Native Cattle	May 5	2005	Cameron
R. microplus	Native Cattle	Feb 1	2005	Cameron
R. microplus	Native Cattle	Mar 7	2007	Cameron
R. microplus	Native Cattle	Jan 7	2005	Cameron
R. microplus	Native Cattle	Aug 3	2004	Hidalgo
R. microplus	Native Cattle	May 5	2008	Hidalgo
R. microplus	Native Cattle	May 6, 19, Jun 8, 15, 29, Jul 13	2009	Starr
R. microplus	Native Cattle	Apr 29	2009	Starr
R. microplus	Native Cattle	Apr 29, Jun 8	2009	Starr
R. microplus	Native Cattle	Apr 15 & 20	2009	Starr
R. microplus	Native Cattle	Jun 8	2009	Starr

R. microplus	Native Cattle	Jun 24	2005	Hidalgo
R. microplus	Native Cattle	Apr 15	2009	Starr
R. microplus	Native Cattle	Jul 21	2009	Starr
R. microplus	Native Cattle	Apr 6	2009	Starr
R. microplus	Native Cattle	Apr 9, 24, May 8, Jun 8, 11, 18, 29	2009	Starr
R. microplus	Native Cattle	Apr 15	2009	Starr
R. microplus	Native Cattle	May 4	2009	Starr
R. microplus	Native Cattle	Jun 25	2009	Starr
R. microplus	Native Cattle	May 13, Jun 25, Jul 27	2009	Starr
R. microplus	Native Cattle	May 19, 29, Jul 13, 27	2009	Starr
R. microplus	Native Cattle	May 19	2009	Starr
R. microplus	Native Cattle	Apr 12, May 12, 26	2005	Starr
R. microplus	Native Cattle	Apr 29	2005	Starr
R. microplus	Native Cattle	Feb 27	2009	Starr
R. microplus	Native Cattle	Jul 2	2009	Starr
R. microplus	Native Cattle	Nov 26	2008	Starr
R. microplus	Native Cattle	Mar 24	2009	Starr
R. microplus	Native Cattle	Mar 27	2009	Starr
R. microplus	Native Cattle	May 1, 4	2009	Starr
R. microplus	Native Cattle	Feb 27	2009	Starr
R. microplus	Native Cattle	Mar 4	2009	Starr
R. microplus	Native Cattle	Dec 19	2008	Starr
R. microplus	Native Cattle	Jan 16	2009	Starr
R. microplus	Native Cattle	Feb 3	2009	Starr
R. microplus	Native Cattle	Feb 27	2009	Starr
R. microplus	Native Cattle	Mar 27, Apr 10, May 4, Jul 8, Aug 13	2009	Starr
R. microplus	Native Cattle	Nov 17	2008	Starr
R. microplus	Native Cattle	Nov 13	2008	Starr
R. microplus	Native Cattle	Nov 25	2008	Starr
R. microplus	Native Cattle	Oct 31, Nov 7	2008	Starr
R. microplus	Native Cattle	Dec 5	2008	Starr
R. microplus	Native Cattle	Dec 19	2008	Starr
R. microplus	Native Cattle	Feb 5	2009	Starr
R. microplus	Native Cattle	Feb 11	2009	Starr
R. microplus	Native Cattle	Dec 9	2008	Starr
R. microplus	Native Cattle	Jun 8 & 18	2009	Starr
R. microplus	Native Cattle	Dec 19	2008	Starr
R. microplus	Native Cattle	Jul 20, 21, Aug 6, 19	2009	Starr
R. microplus	Native Cattle	Apr 3	2009	Starr
R. microplus	Native Cattle	Jun 19	2008	Starr

R. microplus	Native Cattle	Jul 25	2008	Starr
R. microplus	Native Cattle	Mar 13	2009	Starr
R. microplus	Native Cattle	Apr 2	2009	Starr
R. microplus	Native Cattle	Jun 26	2008	Zapata
R. microplus	Native Cattle	Apr 10, 15, 24, May 1, 8, 29, Jun 8, 15, 18	2009	Starr
R. microplus	Native Cattle	Jun 26	2008	Zapata
R. microplus	Native Cattle	Apr 20, 22, May 1, Jul 16	2009	Zapata
R. microplus	Native Cattle	Apr 15, 22, May 1, 14	2009	Zapata
R. microplus	Native Cattle	Apr 6, 15, 22, May 8	2009	Zapata
R. microplus	Native Cattle	Oct 22	2008	Starr
R. microplus	Native Cattle	Mar 13	2009	Starr
R. microplus	Native Cattle	Oct 27	2008	Zapata
R. microplus	Native Cattle	Nov 5	2008	Zapata
R. microplus	Cattle and Deer	Oct 31	2008	Starr
R. microplus	Native Cattle	Oct 17	2008	Starr
R. microplus	Native Cattle	Nov 13	2008	Starr
R. microplus	Native Cattle	Feb 17	2009	Starr
R. microplus	Native Cattle	Mar 4	2009	Starr
R. microplus	White-tailed Deer	Mar 3	2009	Starr
R. microplus	Native Cattle	Oct 24	2008	Starr
R. microplus	White-tailed Deer	Oct 24	2008	Starr
R. microplus	Native Cattle	Nov 5	2008	Zapata
R. microplus	Native Cattle	Aug 6	2004	Starr
R. microplus	Native Cattle	Aug 18	2004	Starr
R. microplus	Native Cattle	Aug 16	2005	Starr
R. microplus	Native Cattle	Jun 14	2007	Starr
R. microplus	Native Cattle	May 4, 14	2009	Zapata
R. microplus	Native Cattle	Sep 6, 20	2005	Starr
R. microplus	Native Cattle	Feb 22	2008	Starr
R. microplus	Native Cattle	Mar 7	2008	Starr
R. microplus	Native Cattle	May 19, Jul 27	2009	Starr
R. microplus	Native Cattle	Jun 16, 25	2009	Zapata
R. microplus	White-tailed Deer	Jun 4	2008	Starr
R. microplus	Native Cattle	May 6,19	2009	Zapata
R. microplus	Native Cattle	Jul 15, 28	2005	Starr
R. microplus	Native Cattle	Apr 6	2005	Starr
R. microplus	Native Cattle	Jun 12	2008	Starr
R. microplus	Native Cattle	Dec 19	2008	Starr
R. microplus	Native Cattle	Jan 15	2009	Starr
R. microplus	Native Cattle	Mar 4	2009	Starr

R. microplus	Native Cattle	Mar 3, Apr 24, May 1, 8, 19, Nov 13, Dec 20	2009	Starr
R. microplus	Native Cattle	May 6	2009	Zapata
R. microplus	Native Cattle	May 6,14	2009	Zapata
R. microplus	Native Cattle	Apr 4, 9, 11	2008	Starr
R. microplus	Native Cattle	May 5	2008	Starr
R. microplus	Native Cattle	Oct 14	2008	Starr
R. microplus	Native Cattle	Oct 27	2008	Starr
R. microplus	Native Cattle	Mar 4	2009	Starr
R. microplus	Native Cattle	Mar 13	2009	Starr
R. microplus	Native Cattle	Mar 18, Jun 25	2009	Starr
R. microplus	Native Cattle	Jul 2	2009	Starr
R. microplus	Native Cattle	Oct 9	2008	Starr
R. microplus	Native Cattle	Oct 27	2008	Starr
R. microplus	Native Cattle	Jan 15	2009	Starr
R. microplus	Native Cattle	Nov 19	2004	Starr
R. microplus	Native Cattle	Dec 8	2004	Starr
R. microplus	Native Cattle	Dec 21, 22	2004	Starr
R. microplus	Native Cattle	May 3, 16	2007	Starr
R. microplus	Native Cattle	Apr 21	2008	Starr
R. microplus	Native Cattle	May 15	2008	Starr
R. microplus	Native Cattle	May 23	2008	Starr
R. microplus	Native Cattle	Mar 18	2009	Starr
R. microplus	Native Cattle	Apr 9, 24, May 19, Nov 13	2009	Starr
R. microplus	White-tailed Deer	Feb 2	2005	Starr
R. microplus	White-tailed Deer	Aug 30	2007	Starr
R. microplus	Native Cattle	Sep 30	2008	Starr
R. microplus	Native Cattle	Nov 21	2008	Starr
R. microplus	Native Cattle	Aug 19	2004	Starr
R. microplus	Native Cattle	May 11, 24	2007	Starr
R. microplus	Native Cattle	Apr 4, 11	2008	Starr
R. microplus	Native Cattle	Jun 20, 22	2007	Starr
R. microplus	Native Cattle	Dec 8, 14	2006	Starr
R. microplus	Native Cattle	Apr 18, May 3	2007	Starr
R. microplus	Native Cattle	Jan 18	2008	Starr
R. microplus	Native Cattle	Jan 18	2008	Starr
R. microplus	Native Cattle	Mar 24	2009	Starr
R. microplus	Native Cattle	Apr 3	2009	Starr
R. microplus	Native Cattle	Mar 27	2008	Starr
R. microplus	White-tailed Deer	Dec 27	2007	Starr
R. microplus	White-tailed Deer	Dec 30	2008	Starr

R. microplus	Native Cattle	Apr 11	2008	Starr
R. microplus	Native Cattle	Sep 15	2004	Starr
R. microplus	Native Cattle	May 15	2007	Starr
R. microplus	Native Cattle	Nov 4	2005	Starr
R. microplus	Native Cattle	May 23	2008	Starr
R. microplus	Native Cattle	Jun 6	2008	Starr
R. microplus	Native Cattle	Jun 24	2008	Starr
R. microplus	Native Cattle	May 13	2005	Starr
R. microplus	Native Cattle	Dec 7	2006	Starr
R. microplus	Native Cattle	Oct 29	2007	Starr
R. microplus	Native Cattle	Aug 17	2007	Starr
R. microplus	Native Cattle	Apr 29, May 3	2005	Starr
R. microplus	Native Cattle	Mar 7, 14	2008	Starr
R. microplus	Native Cattle	Nov 28, Dec 1	2005	Starr
R. microplus	Native Cattle	Jan 16	2006	Starr
R. microplus	Native Cattle	Sep 15	2003	Starr
R. microplus	Native Cattle	Nov 14	2007	Starr
R. microplus	Native Cattle	Jan 31	2008	Starr
R. microplus	Native Cattle	Feb 15	2008	Starr
R. microplus	Mexico Cattle	Nov 15	2007	Starr
R. microplus	Native Cattle	Jun 15, 28	2007	Starr
R. microplus	Native Cattle	May 12	2006	Zapata
R. microplus	Native Cattle	Dec 1, 8	2003	Zapata
R. microplus	Native Cattle	Apr 21	2004	Zapata
R. microplus	Native Cattle	Jul 23	2004	Zapata
R. microplus	Native Cattle	Aug 3	2004	Zapata
R. microplus	Native Cattle	Feb 18	2005	Zapata
R. microplus	Native Cattle	Jun 15	2005	Zapata
R. microplus	White-tailed Deer	May 7	2008	Zapata
R. microplus	Native Cattle	Oct 27	2004	Zapata
R. microplus	Native Cattle	Sep 22	2004	Zapata
R. microplus	Native Cattle	Apr 6	2005	Zapata
R. microplus	Native Cattle	May 6	2005	Zapata
R. microplus	Native Cattle	Jun 15	2005	Zapata
R. microplus	Native Cattle	Jun 19	2008	Zapata
R. microplus	Native Cattle	Oct 6, 8	2004	Zapata
R. microplus	Native Cattle	Mar 13	2008	Starr
R. microplus	Native Cattle	Mar 27	2008	Starr
R. microplus	Native Cattle	Apr 15	2008	Starr
R. microplus	Native Cattle	Apr 23	2008	Starr

R. microplus	Native Cattle,Horse	Feb 20, 28	2008	Starr
R. microplus	Native Cattle	Jun 26	2008	Zapata
R. microplus	Native Cattle	Jul 11	2008	Zapata
R. microplus	Native Cattle	Aug 5	2003	Zapata
R. microplus	Native Cattle	Sep 11	2003	Zapata
R. microplus	Native Cattle	May 13	2004	Zapata
R. microplus	Native Cattle	May 17	2004	Zapata
R. microplus	Native Cattle	Jul 14	2004	Zapata
R. microplus	Native Cattle	Jul 8	2009	Starr
R. microplus	Native Cattle	Jun 3	2005	Zapata
R. microplus	Native Cattle	Mar 14	2005	Starr
R. microplus	Native Cattle	Feb 29	2008	Starr
R. microplus	Native Cattle	Mar 7	2008	Starr
R. microplus	Native Cattle	Sep 27	2003	Zapata
R. microplus	Native Cattle	Oct 22	2004	Zapata
R. microplus	Native Cattle	Mar 10, 25	2005	Starr
R. microplus	Native Cattle	Jun 26	2006	Starr
R. microplus	Native Cattle	Nov 5	2009	Starr
R. microplus	Native Cattle	Mar 24	2008	Starr
R. microplus	Native Cattle	Dec 8	2003	Zapata
R. microplus	Native Cattle	May 15	2008	Zapata
R. microplus	Native Cattle	Nov 14	2003	Zapata
R. microplus	Native Cattle	Jan 7	2005	Zapata
R. microplus	Native Cattle	Mar 22	2005	Zapata
R. microplus	Native Cattle	Mar 4	2009	Zapata
R. microplus	Native Cattle	Feb 3	2009	Zapata
R. microplus	Native Cattle	Aug 18	2003	Zapata
R. microplus	Native Cattle	Sep 22	2003	Zapata
R. microplus	Native Cattle	Apr 25, 29	2008	Zapata
R. microplus	Native Cattle	May 6	2009	Zapata
R. microplus	Native Cattle	May 19	2009	Zapata
R. microplus	Native Cattle	Feb 6	2009	Zapata
R. microplus	Native Cattle	Oct 17	2008	Zapata
R. microplus	Native Cattle	Oct 24	2008	Zapata
R. microplus	Native Cattle	Dec 2	2003	Zapata
R. microplus	Native Cattle	Dec 21	2005	Zapata
R. microplus	Native Cattle	May 14	2004	Zapata
R. microplus	Native Cattle	May 1	2007	Zapata
R. microplus	Native Cattle	Aug 23	2007	Zapata
R. microplus	Native Cattle	Jul 16	2009	Zapata

R. microplus	Native Cattle	Jun 26, Jul 12	2007	Zapata
R. microplus	Native Cattle	Aug 3	2007	Zapata
R. microplus	Native Cattle	Jul 3	2007	Zapata
R. microplus	Native Cattle	Jun 12	2007	Zapata
R. microplus	Native Cattle	Apr 15	2009	Zapata
R. microplus	Native Cattle	Jun 25	2009	Zapata
R. microplus	Native Cattle	Apr 12	2005	Zapata
R. microplus	Native Cattle	Mar 16	2007	Zapata
R. microplus	Native Cattle	Dec 4	2008	Zapata
R. microplus	Native Cattle	Sep 8	2009	Zapata
R. microplus	Native Cattle	Oct 9	2008	Zapata
R. microplus	Native Cattle	Oct 22	2009	Zapata
R. microplus	Native Cattle	Feb 5	2009	Zapata
R. microplus	Native Cattle	Jun 12	2007	Zapata
R. microplus	Native Cattle	Apr 22	2009	Zapata
R. microplus	Native Cattle	Oct 16	2008	Zapata
R. microplus	White-tailed Deer	Dec 28, Jan 13	09,10	Zapata
R. microplus	Native Cattle	Aug 15	2006	Zapata
R. microplus	Native Cattle	May 1, 3	2007	Zapata
R. microplus	Native Cattle	Jun 8, 21	2006	Zapata
R. microplus	Native Cattle	Aug 16	2006	Zapata
R. microplus	White-tailed Deer	Dec 4	2008	Zapata
R. microplus	Native Cattle	Aug 21	2009	Zapata
R. microplus	Native Cattle	Apr 9	2008	Zapata
R. microplus	Native Cattle	Jun 1	2006	Zapata
R. microplus	Native Cattle	Jun 21	2006	Zapata
R. microplus	White-tailed Deer	Dec 17	2009	Zapata
R. microplus	Native Cattle	Jun 13	2006	Zapata
R. microplus	Native Cattle	Apr 6	2009	Zapata
R. microplus	Native Cattle	Dec 18	2007	Zapata
R. microplus	Native Cattle	Jun 23	2004	Zapata
R. microplus	Native Cattle	Jul 6	2005	Zapata
R. microplus	Native Cattle	Aug 30	2005	Zapata
R. microplus	Native Cattle	Dec 21	2005	Zapata
R. microplus	Native Cattle	Jun 20	2007	Zapata
R. microplus	Native Cattle	Apr 1	2008	Zapata
R. microplus	WTD 1/12; Cat 7/27	Jan 12, Jul 27, Nov 16	2009	Zapata
R. microplus	Native Cattle	May 6, 14, Jun 4, 23, Sep 28	2009	Zapata
R. microplus	Native Cattle	Nov 14	2007	Zapata
R. microplus	White-tailed Deer	Apr 29	2008	Zapata

R. microplus	White-tailed Deer	Dec 9	2009	Zapata
R. microplus	Native Cattle	Jun 17	2004	Zapata
R. microplus	Native Cattle	Aug 6	2009	Zapata
R. microplus	Native Cattle	Apr 15	2008	Zapata
R. microplus	Native Cattle	Jul 14	2005	Zapata
R. microplus	Native Cattle	Nov 20	2009	Zapata
R. microplus	Native Cattle	Feb 28, Mar 4	2005	Zapata
R. microplus	White-tailed Deer	Jan 21	2010	Zapata
R. microplus	White-tailed Deer	Nov 21	2008	Zapata
R. microplus	White-tailed Deer	Dec 17	2009	Zapata
R. microplus	White-tailed Deer	Jan 7	2010	Zapata
R. microplus	Native Cattle	Dec 2	2009	Webb
R. microplus	Native Cattle	Apr 24	2009	Zapata
R. microplus	Native Cattle	May 13	2007	Zapata
R. microplus	Native Cattle	Dec 12	2000	Zapata
R. microplus	Native Cattle	Nov 3	2006	Zapata
R. microplus	Native Cattle	Jun 28	2001	Zapata
R. microplus	Native Cattle	Feb 8	2006	Zapata
R. microplus	Native Cattle	Jun 13	2006	Zapata
R. microplus	Native Cattle	Oct 25	2007	Zapata
R. microplus	Native Cattle	Jun 24	2005	Zapata
R. microplus	Mexico Cattle	May 14	2001	Zapata
R. microplus	Native Cattle	Aug 7	2007	Zapata
R. microplus	Native Cattle	Aug 28	2006	Zapata
R. microplus	Native Cattle	Aug 22	2002	Zapata
R. microplus	Native Cattle	Jun 29	2005	Zapata
R. microplus	Native Cattle	Feb 7	2005	Zapata
R. microplus	Native Cattle	Dec 2,17	2009	Zapata
R. microplus	Native Cattle	Nov 9	2004	Zapata
R. microplus	Native Cattle	Jul 11	2000	Zapata
R. microplus	White-tailed Deer	Dec 6, 26, Jan 8	2007	Zapata
R. microplus	Native Cattle	Apr 14	2000	Zapata
R. microplus	Native Cattle	Mar 24	2000	Zapata
R. microplus	Native Cattle	Apr 20	2009	Zapata
R. microplus	Native Cattle	Jul 3	2000	Zapata
R. microplus	Native Cattle	May 1, 6	2009	Zapata
R. microplus	Native Cattle	Apr 27	2001	Zapata
R. microplus	Native Cattle	May 29	2009	Zapata
R. microplus	Native Cattle	Jun 19	2008	Zapata
R. microplus	Native Cattle	Jul 13	2009	Zapata

R. microplus	Native Cattle	Apr 19	2000	Zapata
R. microplus	Native Cattle	Oct 25	2004	Zapata
R. microplus	Native Cattle	Jul 13	2000	Zapata
R. microplus	Native Cattle	Nov 14	2006	Zapata
R. microplus	Native Cattle	Dec 5	2006	Zapata
R. microplus	Native Cattle	Jun 25	2005	Webb
R. microplus	Native Cattle	Oct 19	2007	□Ķ□□□
R. microplus	Native Cattle	Jan 2	2008	Webb
R. microplus	Native Cattle	Jun 29	2005	Webb
R. microplus	Native Cattle	Jun 6	2005	Webb
R. microplus	Native Cattle	Oct 31	2007	Webb
R. microplus	Mexico Cattle	Mar 10	2008	Webb
R. microplus	Native Cattle	Aug 22, Sep 13	2006	Webb
R. microplus	Native Cattle	May 7	2003	Webb
R. microplus	Native Cattle	May 7, 12	2004	Webb
R. microplus	Native Cattle	Jun 26	2006	Webb
R. microplus	Native Cattle	Jul 10	2006	Webb
