# Supplementary material for: Invasive potential of cattle fever ticks in the southern United States
Source: Parasit Vectors. 2014 Apr 17;7:189. doi: 10.1186/1756-3305-7-189 (PMC4021724; doi:10.1186/1756-3305-7-189)

Additional file 3 – An example of spatial selection of persistent occurrences in *Rhipicephalus microplus*

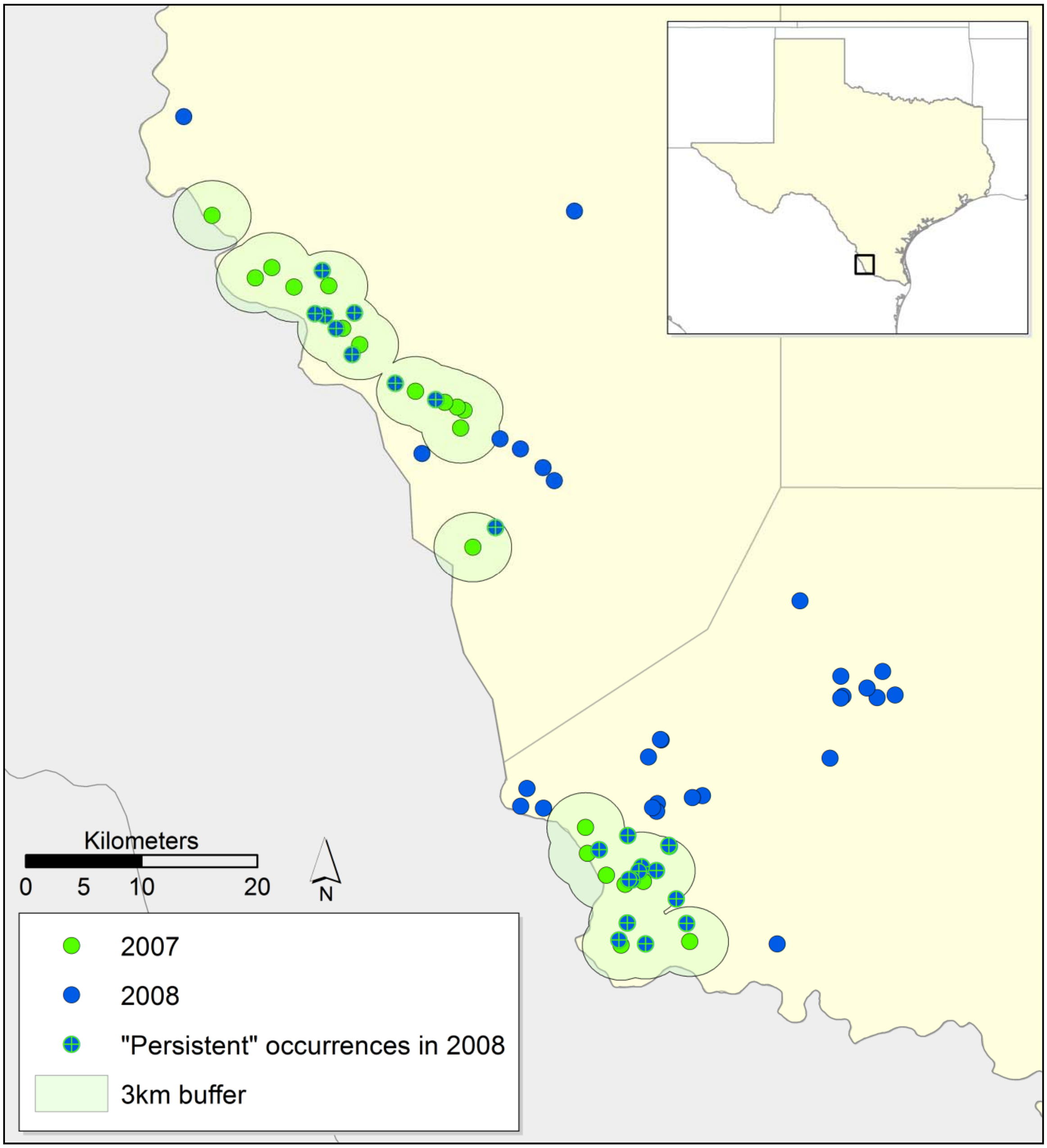

Supplement: Additional file 3 — An example of spatial selection of persistent occurrences in Rhipicephalus microplus. [file 1756-3305-7-189-S3.pdf]
