# Supplementary material for: Invasive potential of cattle fever ticks in the southern United States
Source: Parasit Vectors. 2014 Apr 17;7:189. doi: 10.1186/1756-3305-7-189 (PMC4021724; doi:10.1186/1756-3305-7-189)
Supplement: Additional file 4 — Analysis of Molecular Variance (AMOVA). [file 1756-3305-7-189-S4.pdf]

Additional file 4 – Analysis of Molecular Variance (AMOVA)

Our goal was to estimate spatial extents over which cattle fever tick gene pools are likely able to persist from one generation to the next. We used this information to choose the radius of the buffer used to separate our tick occurrences into ALL or PERS groups. In a study of *R. microplus* infestations on cattle, Brizuela et al. [1] observed that, based on peak tick burdens and the non-parasitic stage of the tick, up to four generations per year are possible, so we predicted that tick generations in Texas may take 3-6 months for both *R. microplus* and *R. annulatus* to complete. We defined a "persistent" infestation as one with a gene pool that remains detectable for >6 months with little or no genetic differentiation from the previous generation (F_{ST}<0.05). We used analysis of molecular variance (AMOVA) as described by Excoffier et al. [2] using the software package GenAlEx 6 to perform among-year comparisons for *R. microplus* tick samples from 2008 versus 2009 (the two sequential years for which genetic data were available) [3, 4].

AMOVA produces an estimate of F_{ST} (Phi$_{ST}$), the commonly used index that represents the distribution of allelic diversity across multiple levels of population subdivision. We used genotyping data from 11 microsatellite loci across 14 collections of *R. microplus* from 2008 and 25 collections from 2009 [4]. Statistical significance for F_{ST} is assessed via random permutation of all population samples, and subsequent calculation of Phi$_{ST}$ after each reshuffling step to generate a distribution of Phi$_{ST}$ values. We chose the "Codom-Allelic" randomization method where all alleles at a single locus are shuffled among individuals at random. Comparison of the observed Phi$_{ST}$ values to the distribution of 999 permutations provided significance values with $\alpha = 0.05$.

Preliminary analysis of genotyped tick samples indicated that 3 km was potentially the maximum distance at which cattle ticks are genetically admixed and subpopulations approach panmixia. Thus, we formed our hypothesis test based on this assumption:

H_O: Little to no genetic difference exists between collections from 2008 to 2009 that are $\leq$ 3 km apart ($F_{ST} \approx 0$, $\alpha \geq 0.05$)

H_A: Genetic differences among collections from 2008 to 2009 that are $\leq$ 3 km apart do exist ($F_{ST} > 0$, $\alpha \leq 0.05$).

We plotted Phi_{ST} values for among-year pairwise comparisons against corresponding spatial distances (Fig 1). As shown in Figure 1, non-significant Phi_{ST} values are observed up to a distance of ~3 km. Most of the significant pairwise values are relatively small ($Phi_{ST} < 0.05$) as well, and in the range of F_{ST} values observed at seven persistent tick locations in Texas [4] Therefore, we observed Phi_{ST} values near or equal to zero in the majority of among-year comparisons ≤3 km apart. The one outlier had an exceptionally high F_{ST} estimate ($Phi_{ST} = 0.34$; Figure 1). We predict this is the result of human-mediated movement of infested cattle from a distant location that were transported during 2009 to within 3 km of where samples had been collected in 2008.

We elected to use 3 km as a reasonable distance with which to define occurrences spatially as persistent. Initially we performed AMOVA on MLVA data from both *R. microplus* as well as *R. annulatus*. However, genotyped data for *R. annulatus* lack spatial resolution <10 km between pairwise comparisons of collections. So, identification of a spatial threshold of persistence for *R. annulatus* was inconclusive: 3 km was also chosen for *R. annulatus* spatial data, with the assumption that both species of cattle fever tick have similar dispersal capabilities in southern Texas.

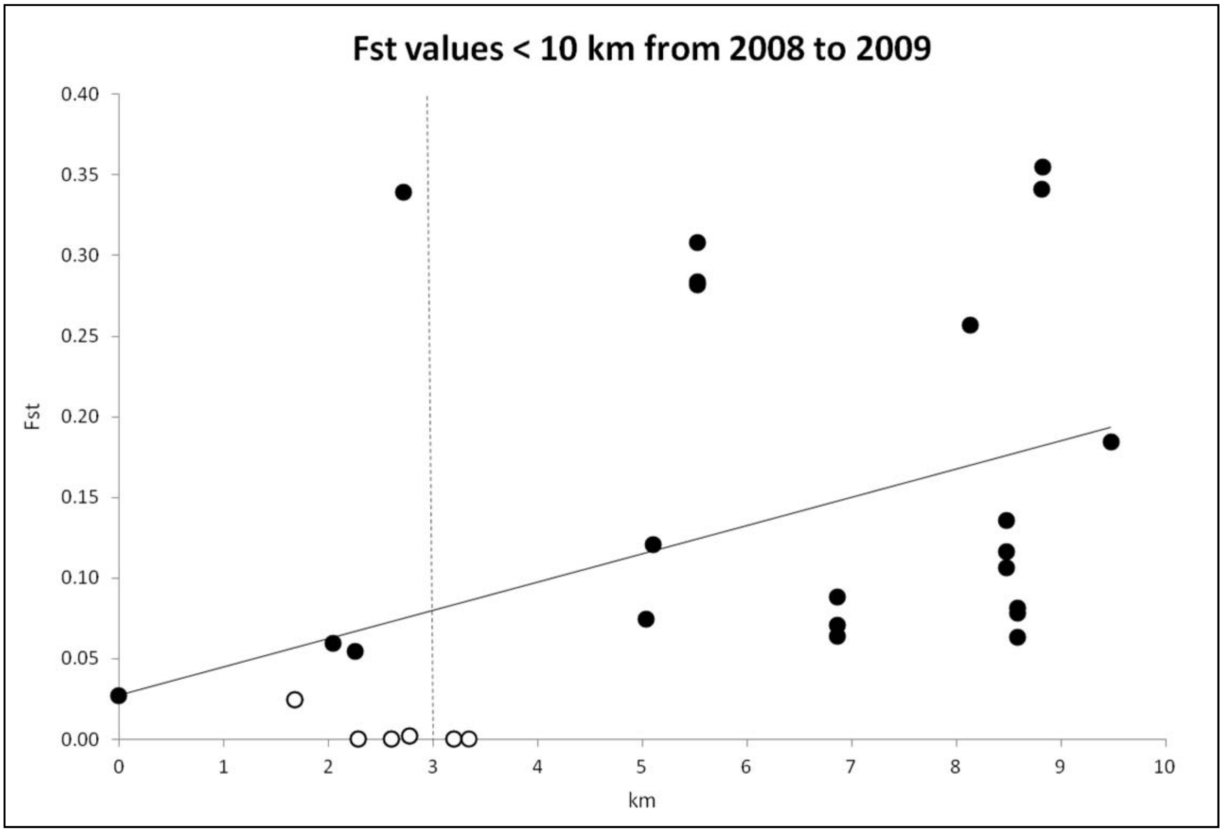

Figure 1. F_{ST} values from 2008-2009 paired samples plotted against spatialdistance (no within-year comparisons are shown). Empty circles represent pairwise comparisons that returned non-significant Phi_{ST} values.

References

1. Brizuela CM, Ortellado CA, Sanchez TI, Osorio O, Walker AR: **Formulation of integrated control of *Boophilus microplus* in Paraguay: analysis of natural infestations.** *Veterinary Parasitology* 1996, **63:**95-108.
2. Excoffier L, Smouse PE, Quattro JM: **Analysis of molecular variance inferred from metric distances among DNA haplotypes: application to human mitochondrial DNA restriction data.** *Genetics* 1992, **131:**479-491.
3. Peakall ROD, Smouse PE: **GENALEX 6: genetic analysis in Excel. Population genetic software for teaching and research.** *Molecular Ecology Notes* 2006, **6:**288-295.
4. Busch JD, Stone NE, Nera R, Araya-Anchetta A, Lewis J, Hochhalter C, Giles JR, Freeman J, Johnson G, Bodine D, et al: **Widespread movement of invasive cattle fever ticks (Rhipicephalus microplus) in southern Texas lead to shared local infestations on cattle and deer.** *Parasites & Vectors* 2013 (in review).
