# Supplementary material for: Invasive potential of cattle fever ticks in the southern United States
Source: Parasit Vectors. 2014 Apr 17;7:189. doi: 10.1186/1756-3305-7-189 (PMC4021724; doi:10.1186/1756-3305-7-189)
Supplement: Additional file 5 — Climate bias analysis. [file 1756-3305-7-189-S5.pdf]

Additional file 6 – Climate Bias Analysis

Selection of climate factors for inclusion in models involved a combination of qualitative (biology-based) and quantitative (data-based) methods. As noted in previous works, climate has been shown to be the most important factor in the ecological success of *Ixodes* ticks, along with host population dynamics [19, 22, 25-27, 31, 32, 49, 50]. So, we focused on reducing environmental parameters from the 19 bioclimatic variables in the WorldClim database to a suite capable of statistically defining suitable habitat for both *R. microplus* and *R. annulatus* [24, 53]. That is to say, variables showing statistical differences between locations where these ticks are known to be present and absent were chosen as predictors.

Our analysis of climate bias was performed as follows: First, our task was identifying regions of presence and absence. Presence sites were defined by polygons drawn around occurrences of both *R. microplus* (n = 314) and *R. annulatus* (n = 63), respectively, with the addition of a 3 km buffer distance to account for the movement of cattle within pastures (repeated for ALL and PERS datasets). Locations of absence were drawn from areas ≤10 km of the US-Mexico border, which we have termed "S" to denote the sample space (roughly corresponding to the TEQA surveyed by USDA-APHIS mounted patrol inspectors). Climate profiles were assembled by extracting raster layer values within these regions (variation shown in the 19 figures below).

We employed a simple randomization test by creating 99 random replicates of an equivalent number of pixels of areas surrounding all (ALL) and persistent (PERS) occurrences for both tick species taken from within the sample space (S). Summary statistics (i.e. median and interquartile range) were calculated for each replicate, and statistical significance estimated based on $\alpha = 0.05$. Comparison of observed climate distributions of areas surrounding ALL

points to that of 99 randomly selected ones within S were significant across all 19 bioclimatic parameters and, therefore, uninformative. However, when areas surrounding PERS locations are contrasted with the random replicates taken from within areas surrounding ALL points, a select few variables were significantly different. Those that showed significance in median and/or interquartile range were subsequently chosen for the development of spatial predictions (Table 1).

For *R. microplus* they are as follows (bioclimatic parameters given in parentheses):

1. annual mean temperature (Bio 1)
2. mean temperature of driest quarter (Bio 9)
3. min temperature of coldest month
4. mean temperature of wettest quarter
5. mean temperature of coldest quarter
6. precipitation seasonality (coefficient of variation)

For *R. annulatus* they are as follows:

1. annual mean temperature (BIO 1)
2. mean diurnal range (mean of monthly (max temp - min temp)) (BIO 2)
3. temperature seasonality (standard deviation *100)
4. max temperature of warmest month
5. mean temperature of coldest quarter
6. precipitation of wettest quarter

In addition to the variables listed above, we have included the environmental distributions of all 19 WorldClim variables for *R. microplus* and *R. annulatus* within the sample space (shown in the 19 figures below).

Table 1. Summary of P-values from randomization tests. Bioclimatic variables that showed significance are highlighted in gray.

			Bio 1	Bio 2	Bio 3	Bio 4	Bio 5	Bio 6	Bio 7	Bio 8	Bio 9	Bio 10	Bio 11	Bio 12	Bio 13	Bio 14	Bio 15	Bio 16	Bio 17	Bio 18	Bio 19
R. annulatus	All within S	Median	0.02	0.02	0.02	0.02	0.44	0.02	0.02	0.02	0.02	0.02	0.02	0.88	0.02	-	0.06	0.02	0.02	0.02	0.02
		IQR	0.02	0.02	0.02	0.02	0.02	0.02	0.02	0.02	0.02	0.02	0.02	0.02	0.02	0.02	0.02	0.02	0.02	0.02	0.02
	Persistent within All	Median	0.02	0.02	-	0.04	0.04	0.02	0.14	0.36	0.36	0.14	0.02	0.02	0.06	0.72	0.12	0.02	0.16	0.28	0.24
		IQR	0.02	0.02	0.08	0.02	0.02	0.22	0.02	0.02	0.02	0.02	0.02	0.24	0.02	0.02	0.02	0.02	0.02	0.02	0.02
R. microplus	All within S	Median	0.1	0.02	-	0.02	0.02	0.02	0.02	0.02	0.02	0.02	0.02	0.02	0.02	-	0.02	0.02	0.02	0.02	0.02
		IQR	0.02	0.02	0.02	0.02	0.02	0.02	0.02	0.02	0.02	0.02	0.02	0.02	0.02	0.02	0.02	0.02	0.02	0.04	0.02
	Persistent within All	Median	0.02	0.2	-	0.2	0.2	0.02	0.2	0.38	0.08	0.2	0.02	0.2	0.2	-	0.2	0.2	0.2	0.2	0.2
		IQR	-	-	0.18	0.18	0.18	0.18	0.18	0.02	0.18	0.16	0.12	0.18	0.18	-	0.02	-	0.2	0.18	0.18

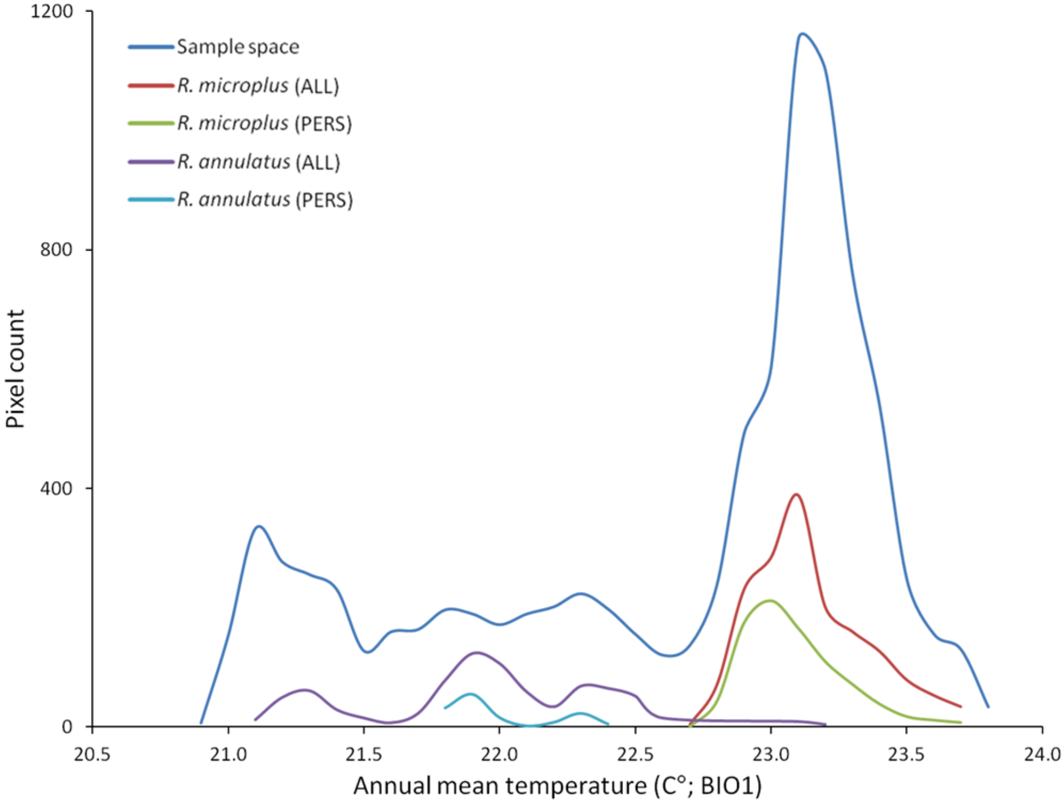

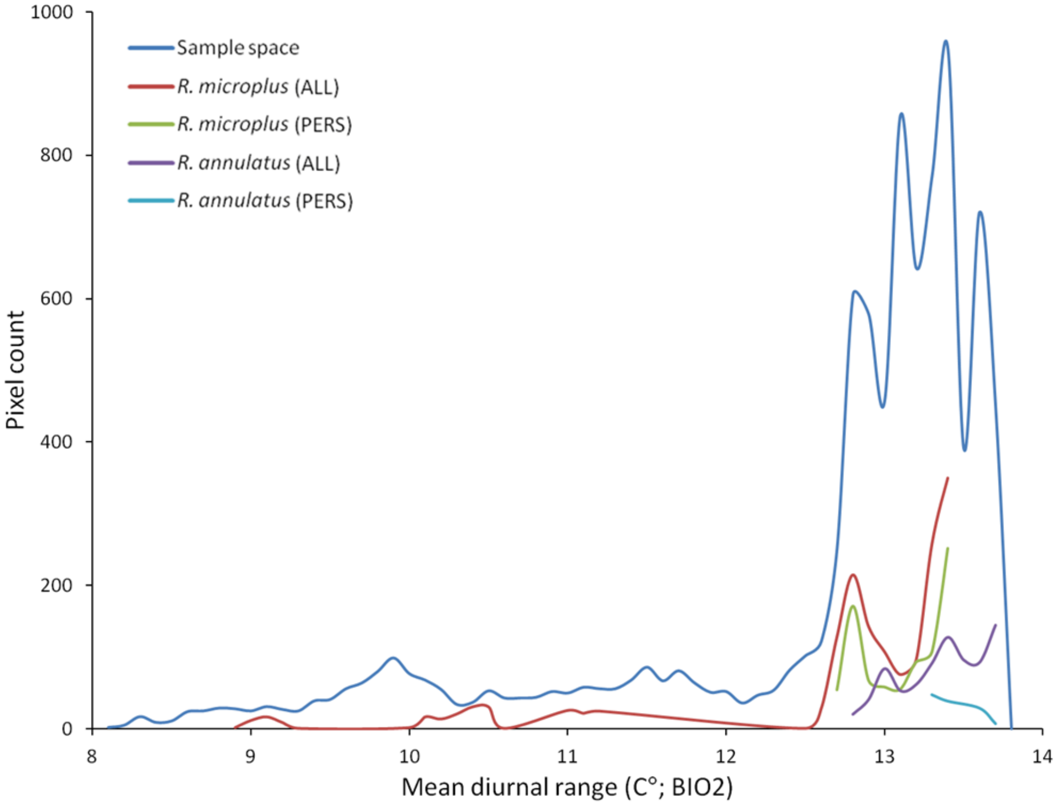
1000
800
600
400
200
0
Pixel count
Sample space
R. microplus (ALL)
R. microplus (PERS)
R. annulatus (ALL)
R. annulatus (PERS)
8
9
10
11
12
13
14
Mean diurnal range (C°; BIO2)

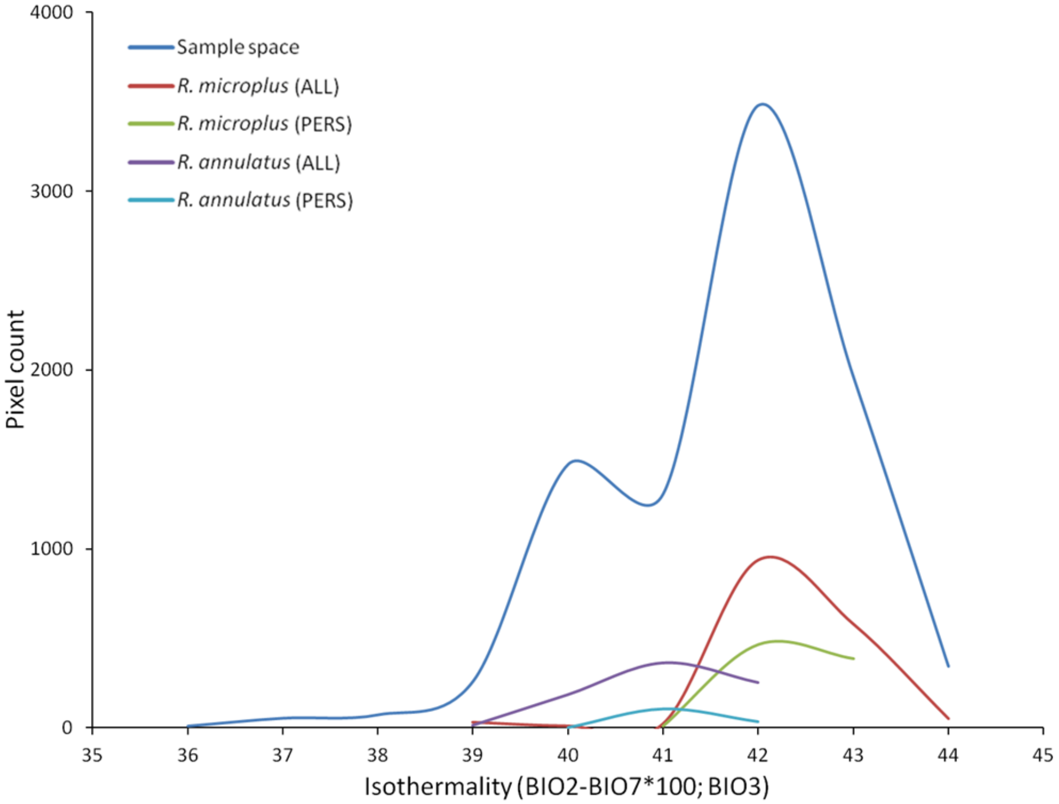
4000
3000
2000
1000
0
Pixel count
Sample space
R. microplus (ALL)
R. microplus (PERS)
R. annulatus (ALL)
R. annulatus (PERS)
35
36
37
38
39
40
41
42
43
44
45
Isothermality (BIO2-BIO7*100; BIO3)

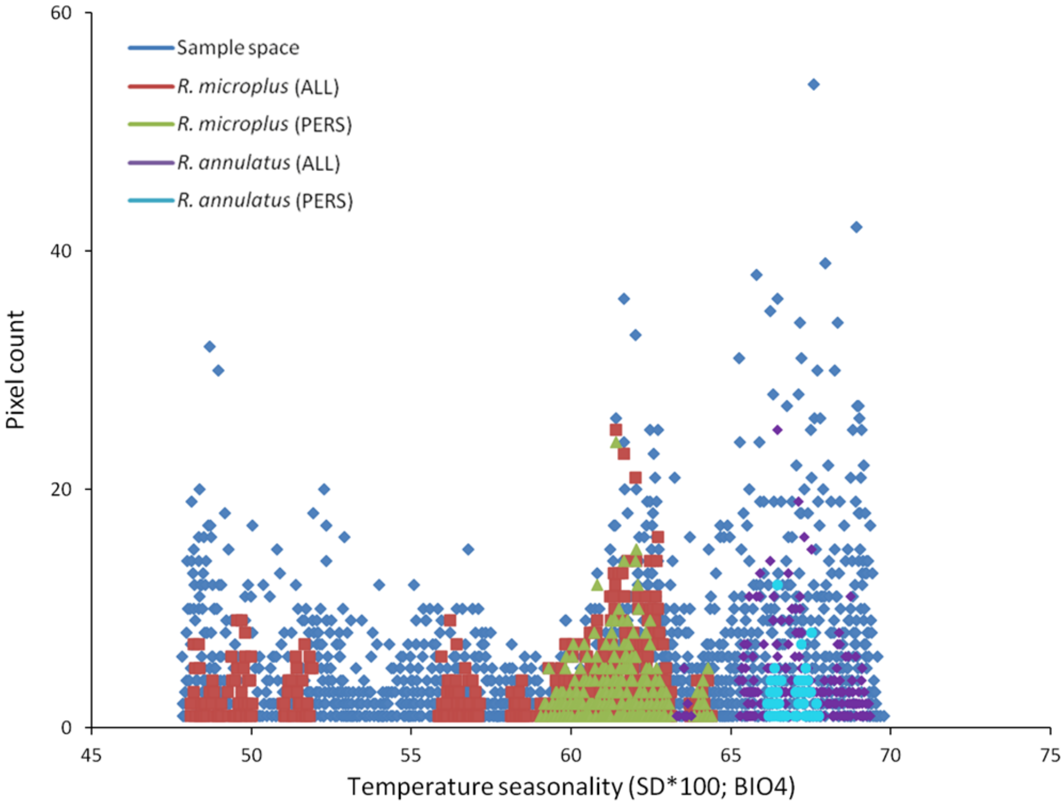

Sample space
R. microplus (ALL)
R. microplus (PERS)
R. annulatus (ALL)
R. annulatus (PERS)
Pixel count
Temperature seasonality (SD*100; BIO4)

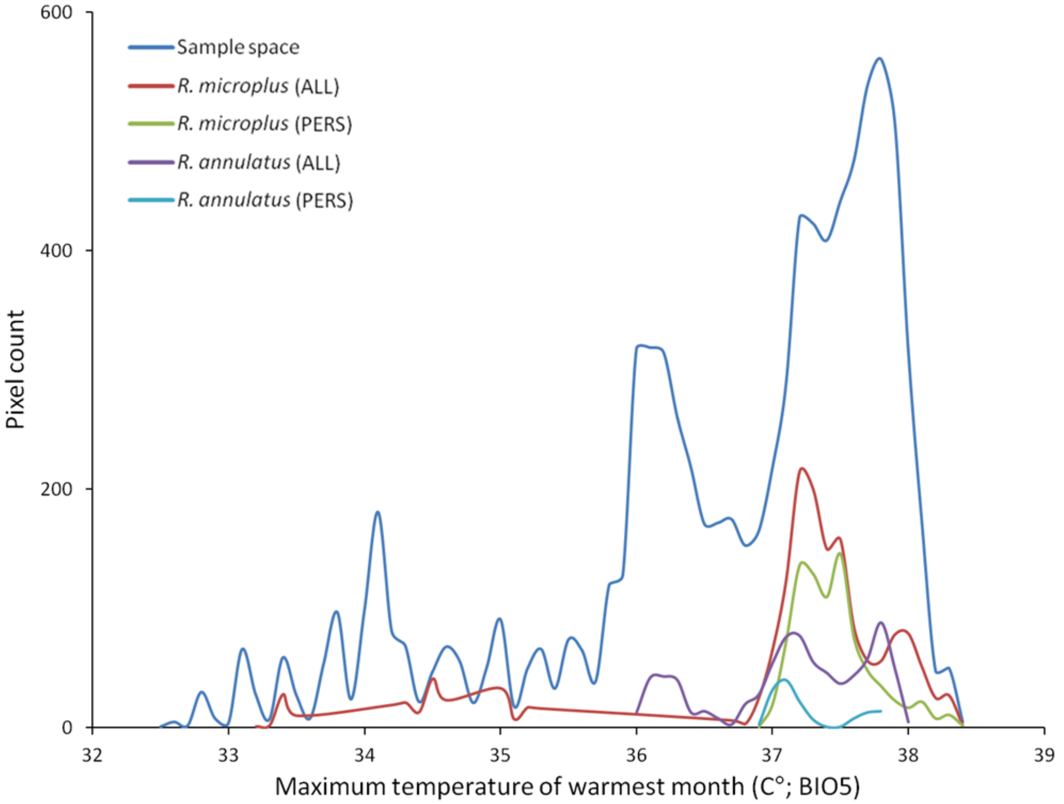

Sample space
R. microplus (ALL)
R. microplus (PERS)
R. annulatus (ALL)
R. annulatus (PERS)
Pixel count
Maximum temperature of warmest month (C°; BIO5)

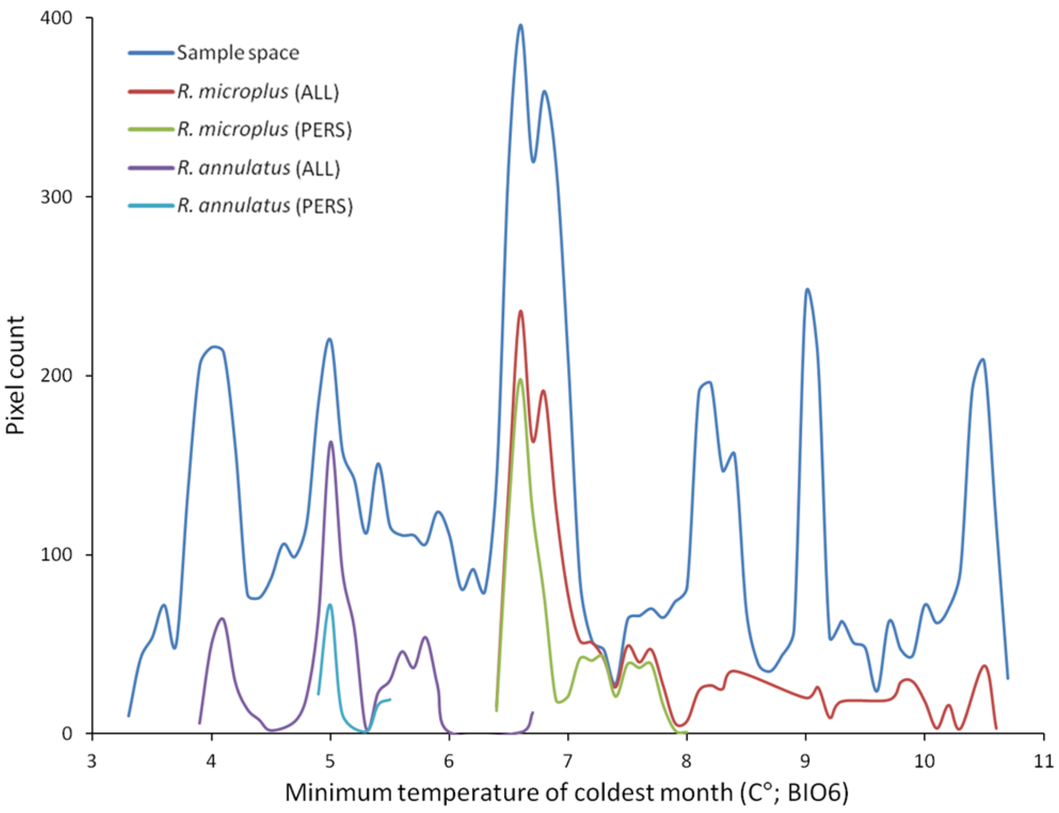

Sample space
R. microplus (ALL)
R. microplus (PERS)
R. annulatus (ALL)
R. annulatus (PERS)
Pixel count
Minimum temperature of coldest month (C°; BIO6)

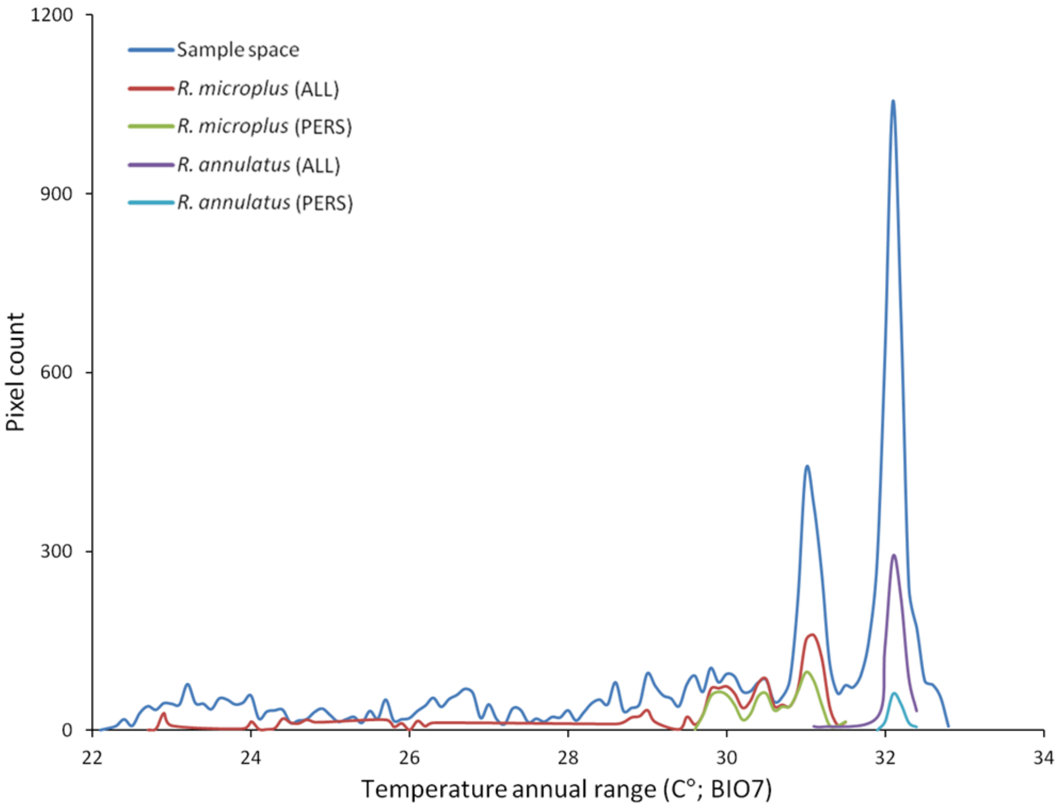

Sample space
R. microplus (ALL)
R. microplus (PERS)
R. annulatus (ALL)
R. annulatus (PERS)
Pixel count
Temperature annual range (C°; BIO7)

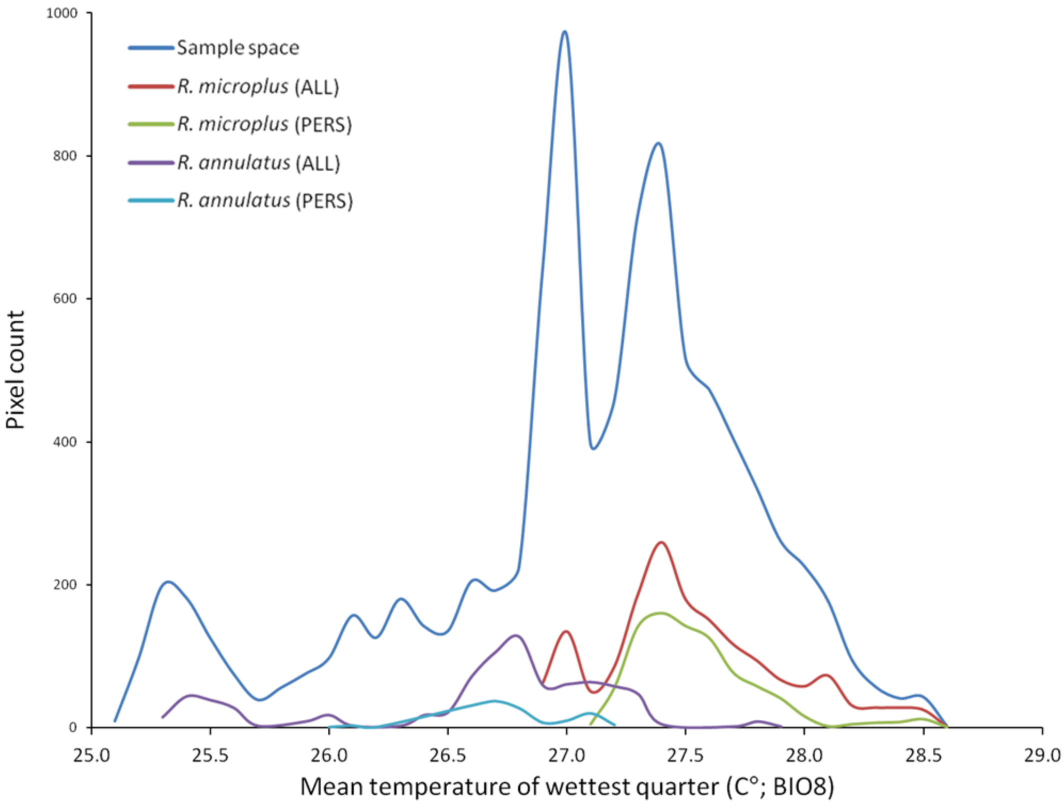
1000
800
600
400
200
0
Pixel count
Sample space
R. microplus (ALL)
R. microplus (PERS)
R. annulatus (ALL)
R. annulatus (PERS)
25.0
25.5
26.0
26.5
27.0
27.5
28.0
28.5
29.0
Mean temperature of wettest quarter (C°; BIO8)

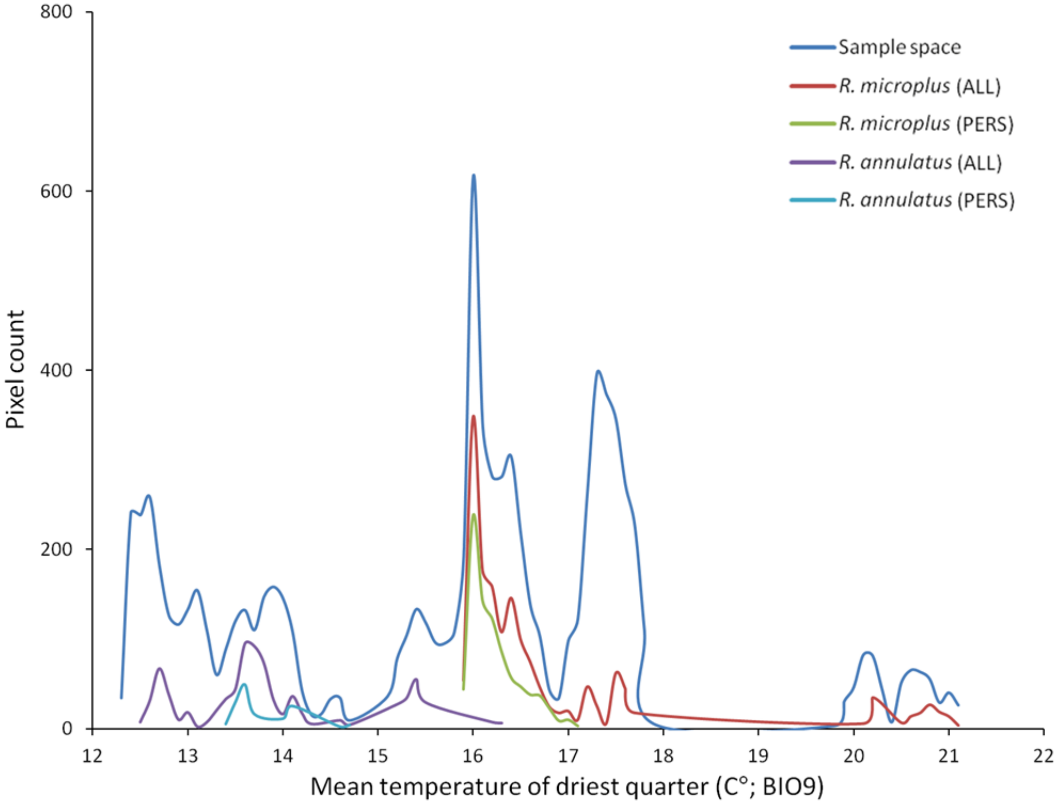
800
600
400
200
0
Pixel count
Sample space
R. microplus (ALL)
R. microplus (PERS)
R. annulatus (ALL)
R. annulatus (PERS)
12
13
14
15
16
17
18
19
20
21
22
Mean temperature of driest quarter (C°; BIO9)

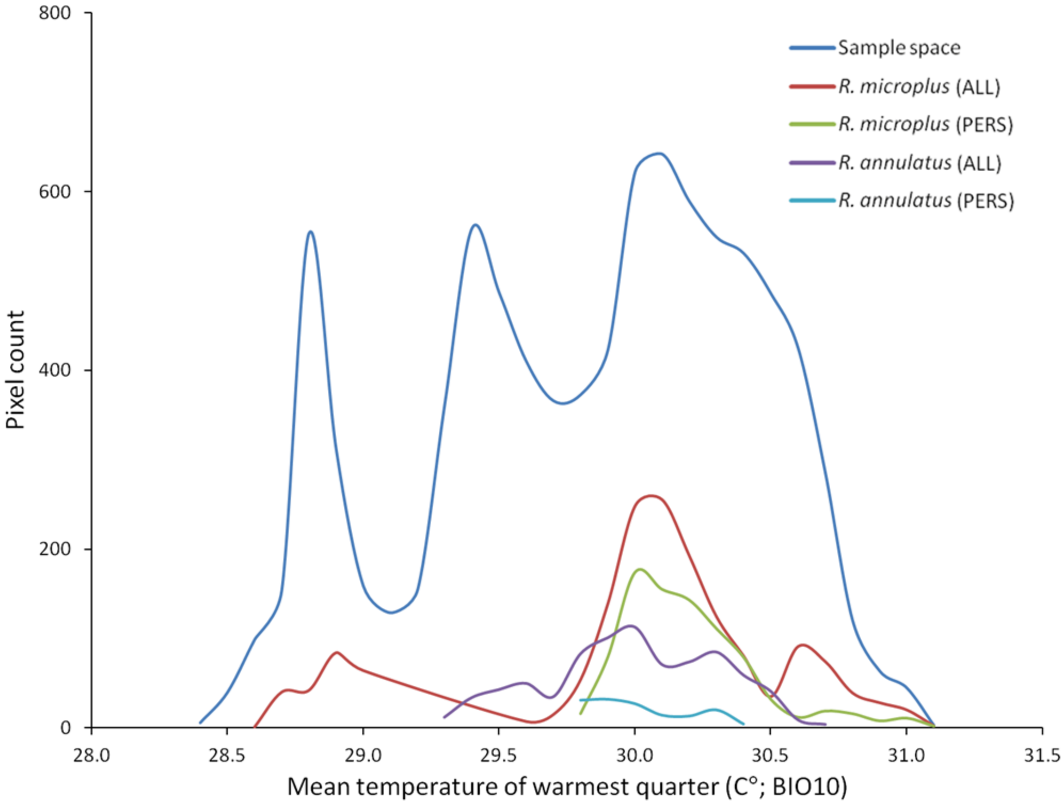
Sample space
R. microplus (ALL)
R. microplus (PERS)
R. annulatus (ALL)
R. annulatus (PERS)
Pixel count
800
600
400
200
0
28.0
28.5
29.0
29.5
30.0
30.5
31.0
31.5
Mean temperature of warmest quarter (C°; BIO10)

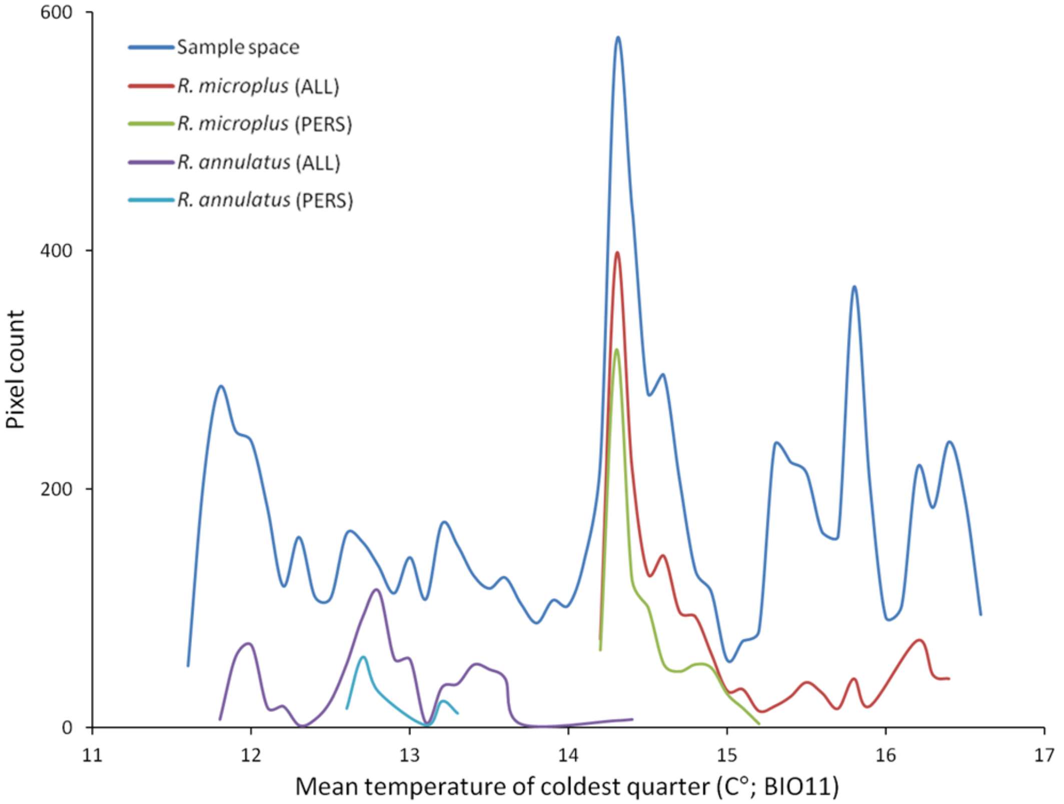
Sample space
R. microplus (ALL)
R. microplus (PERS)
R. annulatus (ALL)
R. annulatus (PERS)
Pixel count
600
400
200
0
11
12
13
14
15
16
17
Mean temperature of coldest quarter (C°; BIO11)

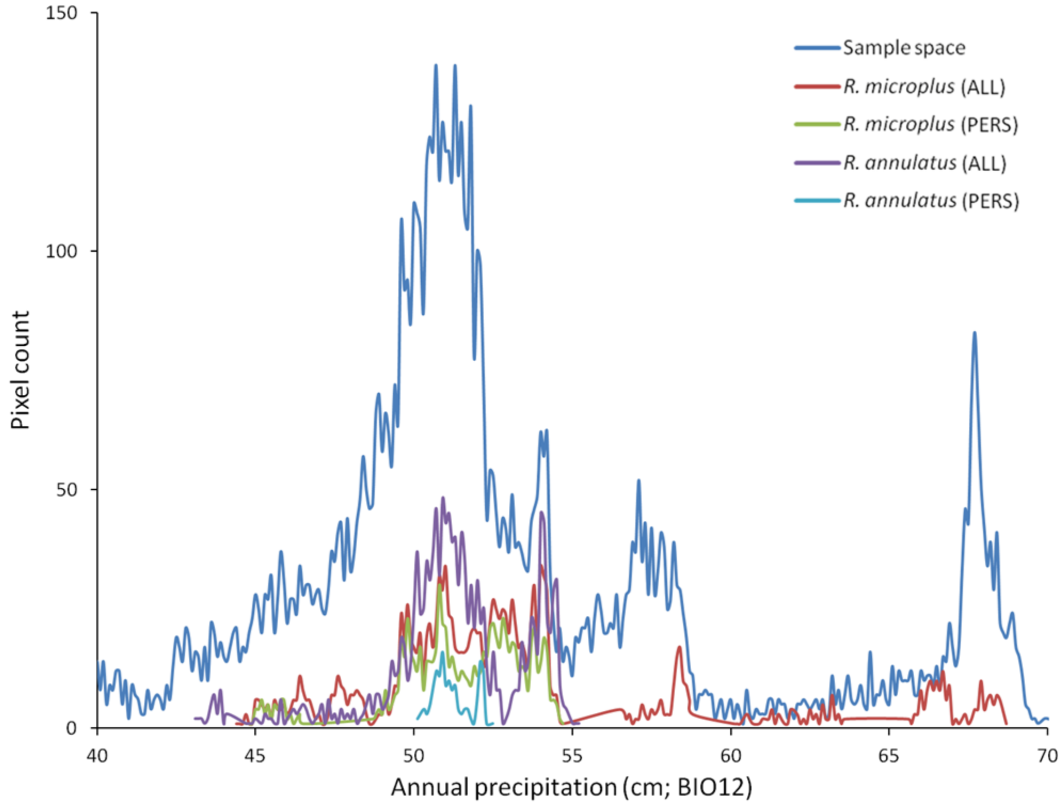
150
100
50
0
40
45
50
55
60
65
70
Pixel count
Annual precipitation (cm; BIO12)
Sample space
R. microplus (ALL)
R. microplus (PERS)
R. annulatus (ALL)
R. annulatus (PERS)

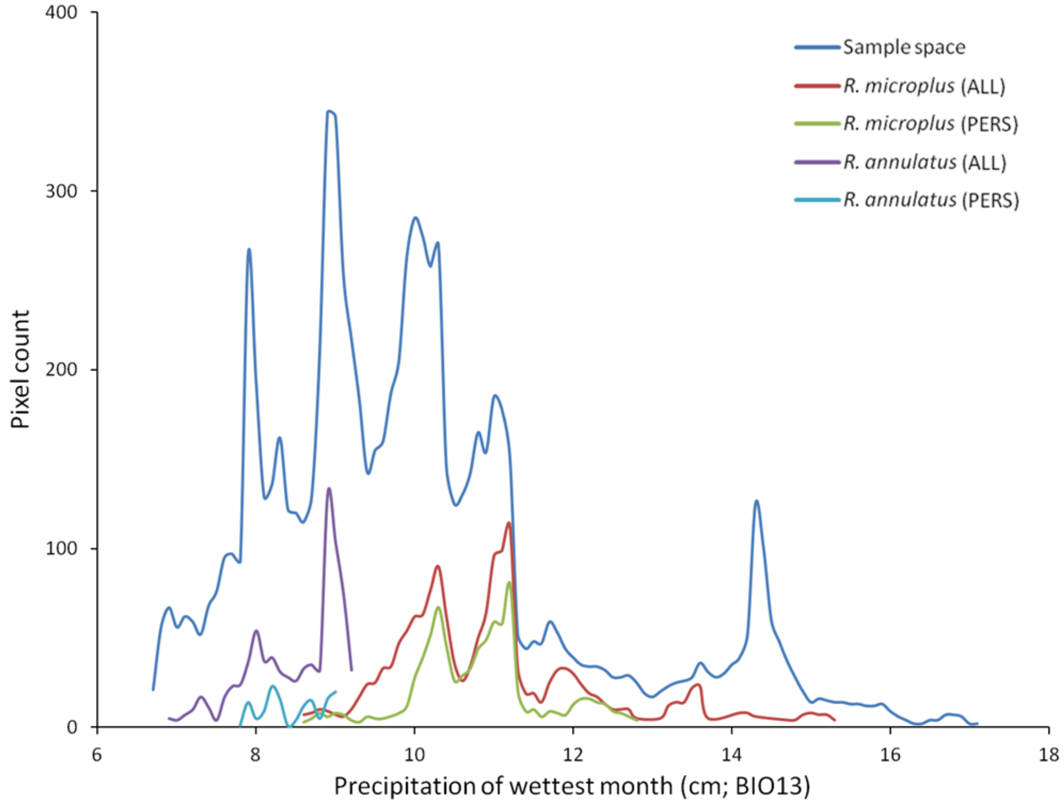
400
300
200
100
0
6
8
10
12
14
16
18
Pixel count
Precipitation of wettest month (cm; BIO13)
Sample space
R. microplus (ALL)
R. microplus (PERS)
R. annulatus (ALL)
R. annulatus (PERS)

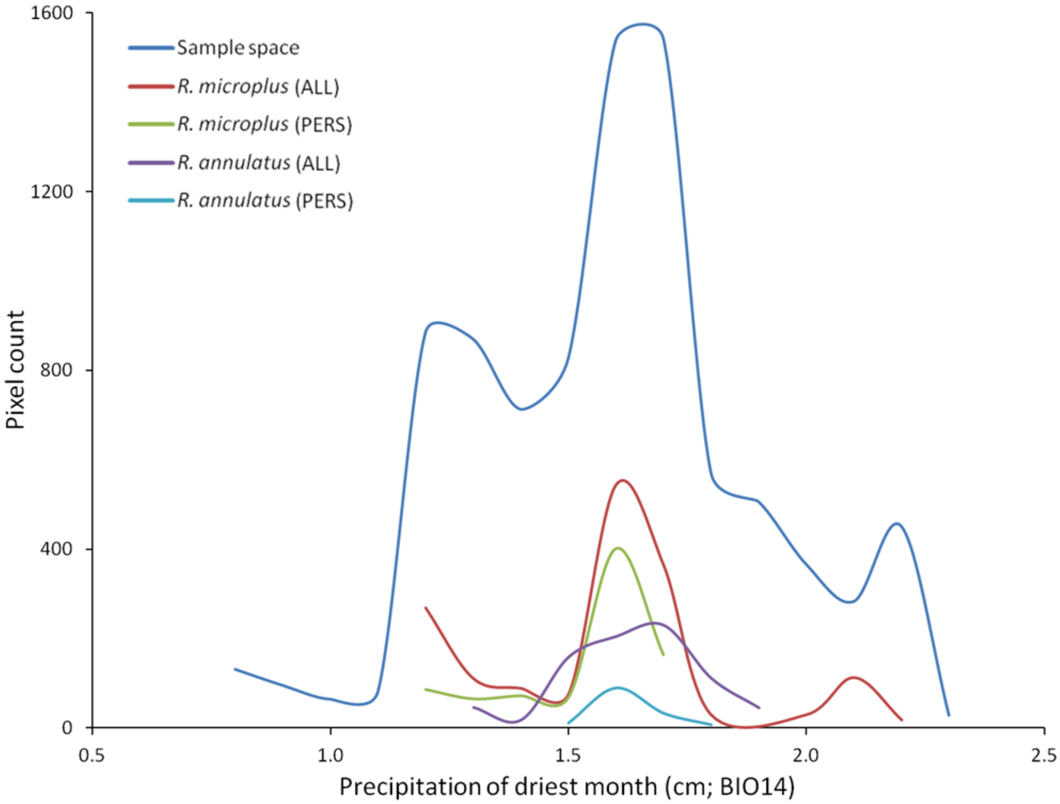
1600
1200
800
400
0
0.5
1.0
1.5
2.0
2.5
Pixel count
Precipitation of driest month (cm; BIO14)
Sample space
R. microplus (ALL)
R. microplus (PERS)
R. annulatus (ALL)
R. annulatus (PERS)

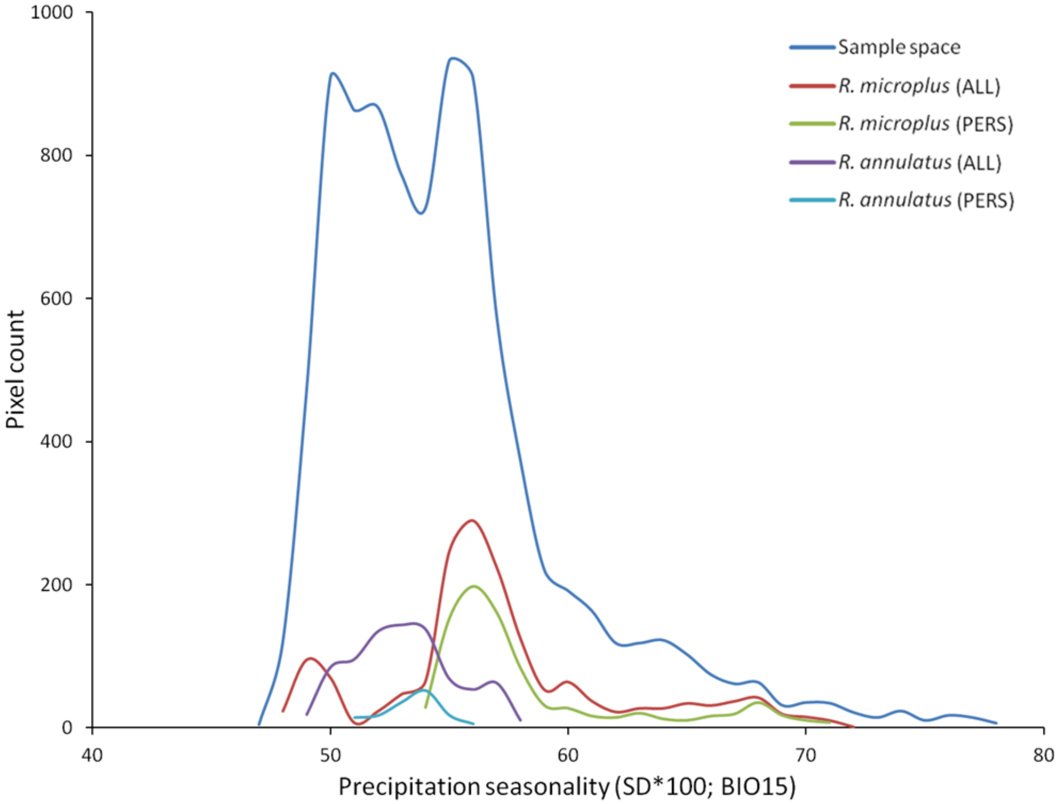
1000
800
600
400
200
0
40
50
60
70
80
Pixel count
Precipitation seasonality (SD*100; BIO15)
Sample space
R. microplus (ALL)
R. microplus (PERS)
R. annulatus (ALL)
R. annulatus (PERS)

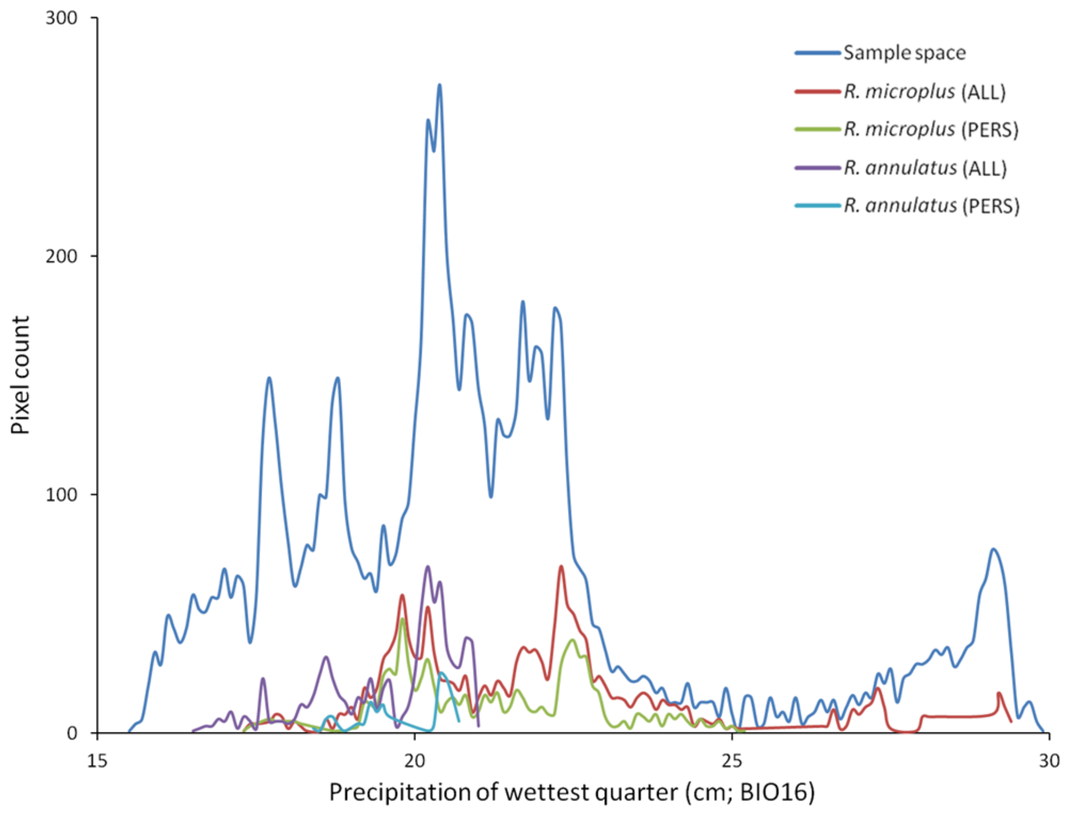

300
200
100
0
15
20
25
30
Pixel count
Precipitation of wettest quarter (cm; BIO16)
Sample space
R. microplus (ALL)
R. microplus (PERS)
R. annulatus (ALL)
R. annulatus (PERS)

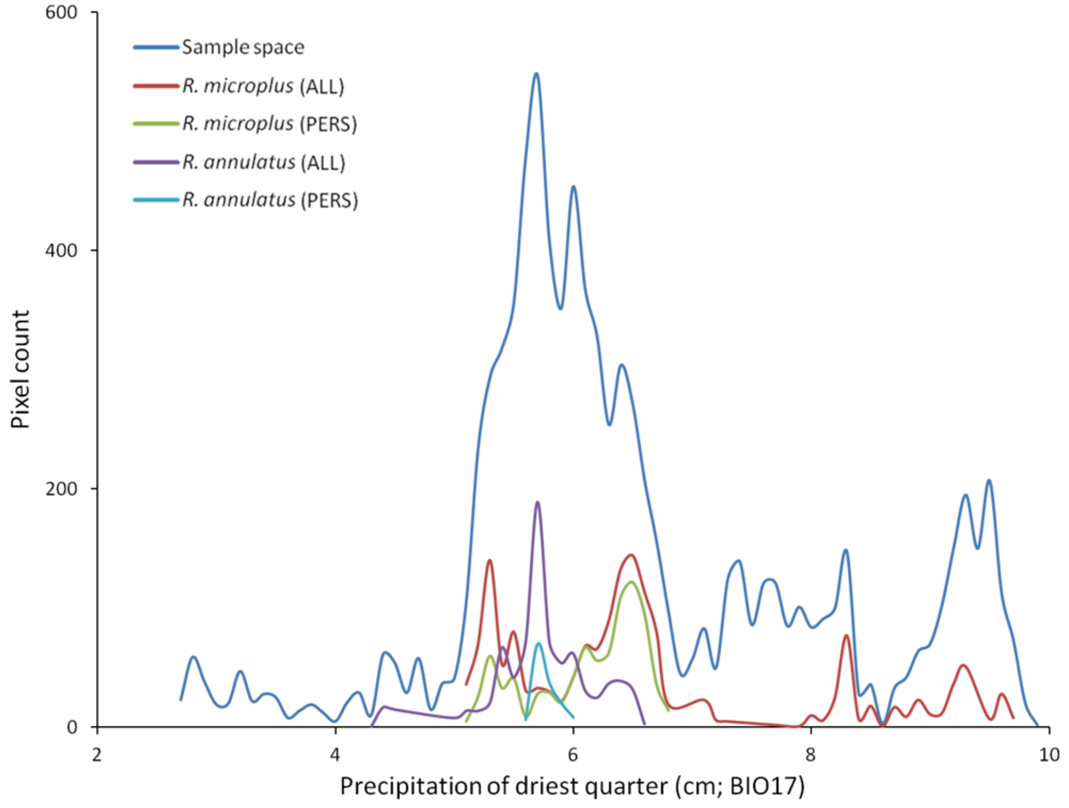

600
400
200
0
2
4
6
8
10
Pixel count
Precipitation of driest quarter (cm; BIO17)
Sample space
R. microplus (ALL)
R. microplus (PERS)
R. annulatus (ALL)
R. annulatus (PERS)

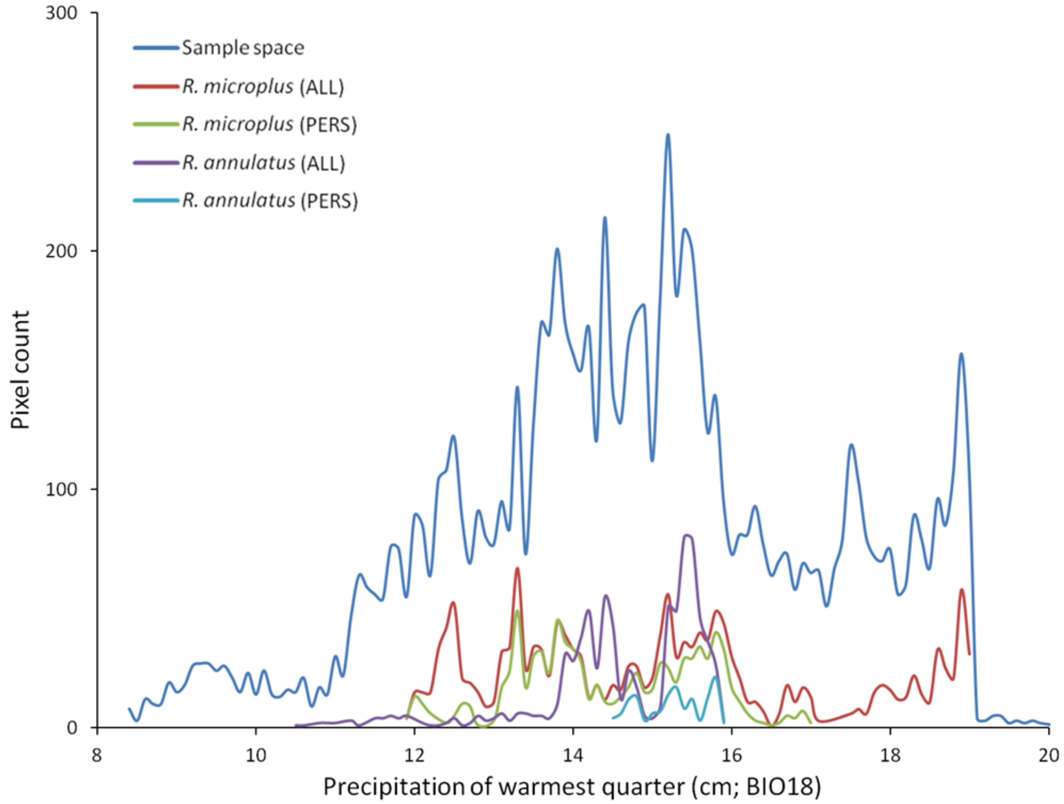

300
200
100
0
8
10
12
14
16
18
20
Pixel count
Precipitation of warmest quarter (cm; BIO18)
Sample space
R. microplus (ALL)
R. microplus (PERS)
R. annulatus (ALL)
R. annulatus (PERS)

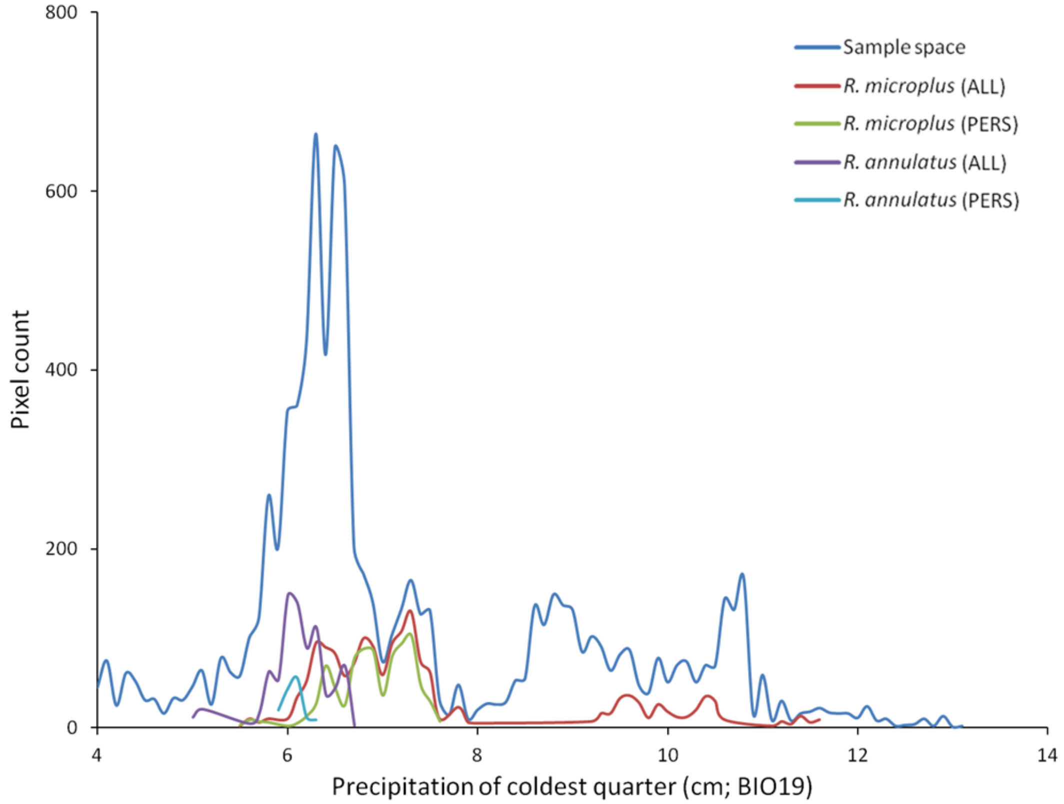

800
600
400
200
0
4
6
8
10
12
14
Pixel count
Precipitation of coldest quarter (cm; BIO19)
Sample space
R. microplus (ALL)
R. microplus (PERS)
R. annulatus (ALL)
R. annulatus (PERS)

References

1. Randolph S: **Ticks and tickoborne disease systems in space and from the space.** *Advances in Parasitology* 2000, **47:**217-243.
2. Teel PD: **Effect of saturation deficit on eggs of *Boophilus annulatus* and *B. microplus* (Acari: Ixodidae).** *Annals of the Entomological Society of America* 1984, **77:**65-68.
3. Estrada-Peña A, Bouattour A, Camicas JL, Guglielmone A, Horak I, Jongejan F, Latif A, Pegram R, Walker AR: **The known distribution and ecological preferences of the tick subgenus *Boophilus* (Acari: Ixodidae) in Africa and Latin America.** *Experimental and Applied Acarology* 2006, **38:**219-235.
4. Davey RB, Cooksey LM, Despins JL: **Survival of larvae of *Boophilus annulatus, Boophilus microplus,* and *Boophilus hybrids* (Acari: Ixodidae) in different temperature and humidity regimes in the laboratory.** *Veterinary Parasitology* 1991, **40:**305-313.
5. Mount GA, Haile DG, Barnard DR, Daniels E: **New version of LSTSIM for computer simulation of *Amblyomma americanum* (Acari: Ixodidae) population dynamics.** *Journal of Medical Entomology* 1993, **30:**843-857.
6. Mount GA, Haile DG, Daniels E: **Simulation of blacklegged tick (Acari: Ixodidae) population dynamics and transmission of *Borrelia burgdorferi*.** *Journal of Medical Entomology* 1997, **34:**461-484.
7. Mount GA, Haile DG, Davey RB, Cooksey LM: **Computer simulation of *Boophilus* cattle tick (Acari: Ixodidae) population dynamics.** *Journal of Medical Entomology* 1991, **28:**223-240.
8. Gaff H, Gross L: **Modeling tick-borne disease: a metapopulation model.** *Bulletin of Mathematical Biology* 2007, **69:**265-288.
9. Wang H-H, Grant WE, Teel PD: **Simulation of climate-host-parasite-landscape interactions: a spatially explicit model for ticks (Acari: Ixodidae).** *Ecological Modelling* 2012, **243:**42-62.
10. Hijmans RJ, Cameron SE, Parra JL, Jones PG, Jarvis A: **Very high resolution interpolated climate surfaces for global land areas.** *International Journal of Climatology* 2005, **25:**1965-1978.
11. Estrada-Peña A, Venzal JM: **High-resolution predictive mapping for *Boophilus annulatus* and *B. microplus* (Acari: ixodidae) in Mexico and Southern Texas.** *Veterinary Parasitology* 2006, **142:**350-358.
