# Supplementary material for: Invasive potential of cattle fever ticks in the southern United States
Source: Parasit Vectors. 2014 Apr 17;7:189. doi: 10.1186/1756-3305-7-189 (PMC4021724; doi:10.1186/1756-3305-7-189)
Supplement: Additional file 6 — Spatial autocorrelation & data rarefaction. [file 1756-3305-7-189-S6.pdf]

Additional file 5 – Spatial Autocorrelation & Data Rarefaction

In light of the clustered nature of our occurrence data, we performed a structural analysis of the orthogonal variance among the subset of six climate variables chosen for the modeling process. This analysis was done using a variogram model made in the Geostatistical Analyst extension of ArcMap 10 [1]. To produce variograms that binned pairwise values with adequate spatial resolution, we set lag size at 0.05 decimal degrees and number of lags to 20 (per ESRI's recommendations considering the distribution of the data).

Variography displays differences in raster layer values between pairs of sampled locations as a function of the distances separating them. To summarize the structure of climate variation across the area surrounding the quarantine zone, we created a shapefile containing 1000 random locations within 10 km of thetick eradication quarantine area, and populated each point with its corresponding raster value from principal component 1. An exponential linear model is then fitted to the variogram, and important metrics such as the nugget, range, and sill calculated, which were used to identify at what distance a variable is no longer correlated in space (spatial lag).

We calculated spatial lag as the range value observed when the variogram model reaches 80% of the sill value. This method estimates the spatial lag as 7 km and 4.5 km for *R. microplus* and *R. annulatus*, respectively. It is important to note that analysis via variogram does not directly calculate a single metric of spatial lag. Rather, this approach displays distances between two points that are capable of producing variances that are 80% of the sill value.

Structural analysis of the orthogonal variance among the subset of six climate variables chosen for the modeling process provided estimates of spatial lag which we used to filter occurrences spatially in the spatial analysis software package, Geospatial Modeling Environment

(GME; [2]). Random subsets were formed by generating a vector grid with cell sizes equal to the spatial lag value. Unique cell identification is assigned to each occurrence point so that only one is chosen per cell (Figure 1). This procedure was repeated 10 times before subsets are extracted in ArcMap according to their assigned random cell ID so that 10 replicate occurrence datasets are formed with each point separated by a distance greater than the spatial lag. Table 1 displays the relative sample sizes after data reduction.

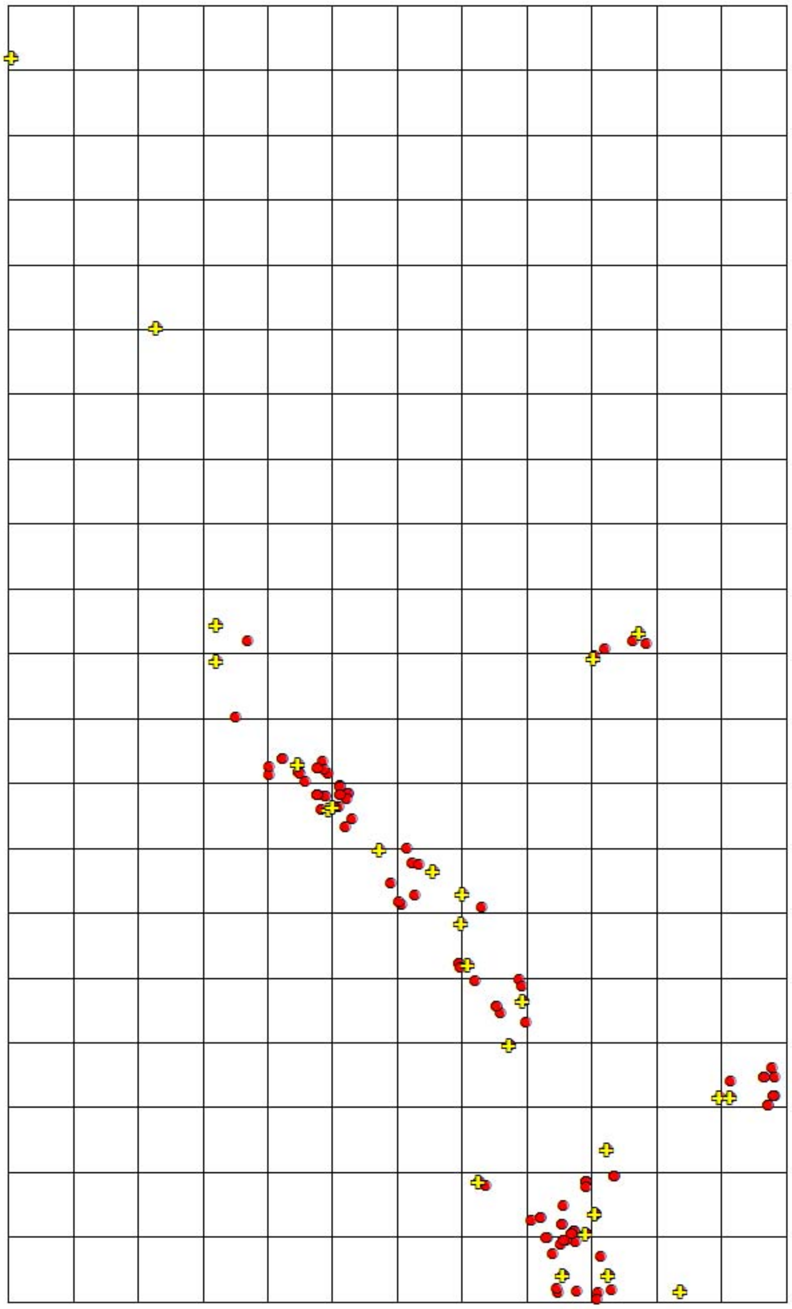

Figure 1. Example of spatial rarefaction of point data where a vector grid with a cell size equal to the spatial lag distance is laid over the occurrences (red circles) and one point per cell is extracted (yellow crosses).

Table 1: Breakdown of sample sizes resulting from data rarefaction. “S” denotes the number of occurrences that occur within the designated sample space (areas near the tick eradication quarantine area) that were subsequently used for the formation of spatial models.

		Original sample size	Spatial errors removed		Sample size of each random subset		Random subsets
			In all	Within “S”	In all	Within “S”	
R. microplus	ALL	347	314	246	60	44	10
	PERS	-	151	129	25	20	10
R. annulatus	ALL	76	63	40	41	27	10
	PERS	-	13	7	13	7	-

References

1. ESRI: **ArcGIS desktop: release 10.** Redlands, CA: Environmental Systems Research Institute; 2011.
2. Beyer H: **Geospatial modelling environment.** Spatial Ecology LLC; 2010.
