# Supplementary material for: Invasive potential of cattle fever ticks in the southern United States
Source: Parasit Vectors. 2014 Apr 17;7:189. doi: 10.1186/1756-3305-7-189 (PMC4021724; doi:10.1186/1756-3305-7-189)
Supplement: Additional file 7 — Algorithm parameters and model summarization. [file 1756-3305-7-189-S7.pdf]

Additional file 7 – Algorithm Parameters and Model Summarization

Methods for estimating the ecological niche of biological phenomena have seen substantial attention in recent years [1-5]. Specifically, the use of presence-only data has evoked extensive discussion on the assumptions that are made when using modelling algorithms that create their own pseudo-absence data [5-9]. Niche estimation methods such as the Genetic Algorithm for Rule-Set Production (GARP) and maximum entropy (Maxent) randomly sample a user-defined background area in the formation of pseudo-absence data with which to build spatial predictions. So, definition of the background landscape in presence-only modelling pursuits is of paramount importance, as adeptly shown by Barve et al. [9, 10]. Elith et al. [9] point out that the background area (i.e. landscape of interest (*L*), as the referred document states) sampled for pseudo-absences is "suggested by the problem and defined by the ecologist".

The sampling scheme set in place by the USDA and APHIS made the definition of the background area (herein referred to as sample space, or "S") a conveniently straightforward one. CFTEP inspectors systematically patrol within the TEQA in search of *R. microplus* or *R. annulatus* presence. So, locations within this thoroughly surveyed region where the tick species have not been observed would naturally serve as an appropriate pseudo-absence, were GARP or Maxent to sample one there. Therefore, we defined our background as the area along the TEQA that is within 10km of the US-Mexico border (roughly equal to the area surveyed by USDA-APHIS inspectors).

GARP is a random-walk process that evolves a predictive rule (e.g. logistic regression, bioclimatic and range rules etc.) with subsequent iterations until minimal improvement in the prediction of independent test data is achieved. Maxent forms model predictions by maximizing

the entropy between the probability distribution of environmental variables at locations of presence to that of the user-selected study area [9, 11-13].

GARP models were built using the 'best subsets' method, which filters through internally created models so that only an accurate subset with low omission and commission error is retained [14, 15]. Maxent models were trained with default parameters intact, except training data allocation was set to 50% and the 'clamping' setting was removed [16]. The clamping parameter inhibits spatial projections from predicting novel environments as suitable. Considering the disequilibrium in both *R. microplus* and *R. annulatus* distributions that result from the eradication effort, use of this default setting would result in spurious spatial prediction. As an example, the reproductive temperature tolerances of both ticks species, independent of ambient humidity, occurs between 20-35°C [17, 18]. The mean temperature tolerances of our observed occurrences exhibit a much more conservative range of tolerance (20-24°C; see Additional file 6). This reduction in range of mean temperature has likely resulted from the artificially confined distribution of both tick species within the quarantine zone.

Spatial models for each of the ten random subsets were converted to binary predictions via the 'lowest presence threshold' (LPT) technique developed by Pearson et al. [19] then summed to produce an amalgamate model containing the agreement in spatial prediction of all 10 random subsets (e.g. a value of 5 means that 5 of the random subset models agree on suitability at a grid cell).

Although we initially explored both algorithms, our goal was to present results of just one for simplicity. Additionally, all models contain a measure of uncertainty, so assessment via independent test data is necessary. So, we performed model validation by regional division of occurrences and subsequent prediction of independent testing points. Tests were developed

within 50 km of occurrence data under a $k - 1$ framework, where $k = 4$ subdivisions of our occurrence data. We then tested models built with all combinations of three sub regions in their predictive accuracy of occurrences within the excluded fourth region (Figure 1; [14]). *R. annulatus* occurrences were highly clustered and prevented acceptable subdivision into independent testing regions, so model testing was performed only with *R. microplus* occurrences.

Initially the area under the curve (AUC) of the receiver operating characteristic (ROC) was developed in World War II for radar signal detection; however it has recently been applied as a standard method for the validation of species distribution models [8, 20]. Questions as to the reliability of traditional AUC have been raised, prompting discussion regarding how this method can be retro-fitted to the SDM field [7, 8, 21, 22]. We calculated partial ROC-AUC ratios using the Partial ROC software package developed by Barve [23], which allows for reliable comparison of model performance between spatial predictions produced by both GARP and Maxent and calculation of statistical significance [22]. AUC ratios were calculated with default parameters; however the omission threshold here was relaxed to 0.9. Maxent performed better than GARP in partial AUC scores across all four testing regions, so results produced by Maxent are used in this study (Table 1).

Table 1. Partial AUC ratios of Maxent and GARP

	Maxent				GARP			
	Reg. 1	Reg. 2	Reg. 3	Reg. 4	Reg. 1	Reg. 2	Reg. 3	Reg. 4
Mean	1.60	1.59	1.57	1.71	1.23	1.56	1.22	1.42
Standard Deviation	0.05	0.04	0.08	0.04	0.17	0.16	0.19	0.20
Number of replicates ≤ 1	0	0	0	0	103	7	342	89
P value	0	0	0	0	0.010	0.001	0.034	0.009

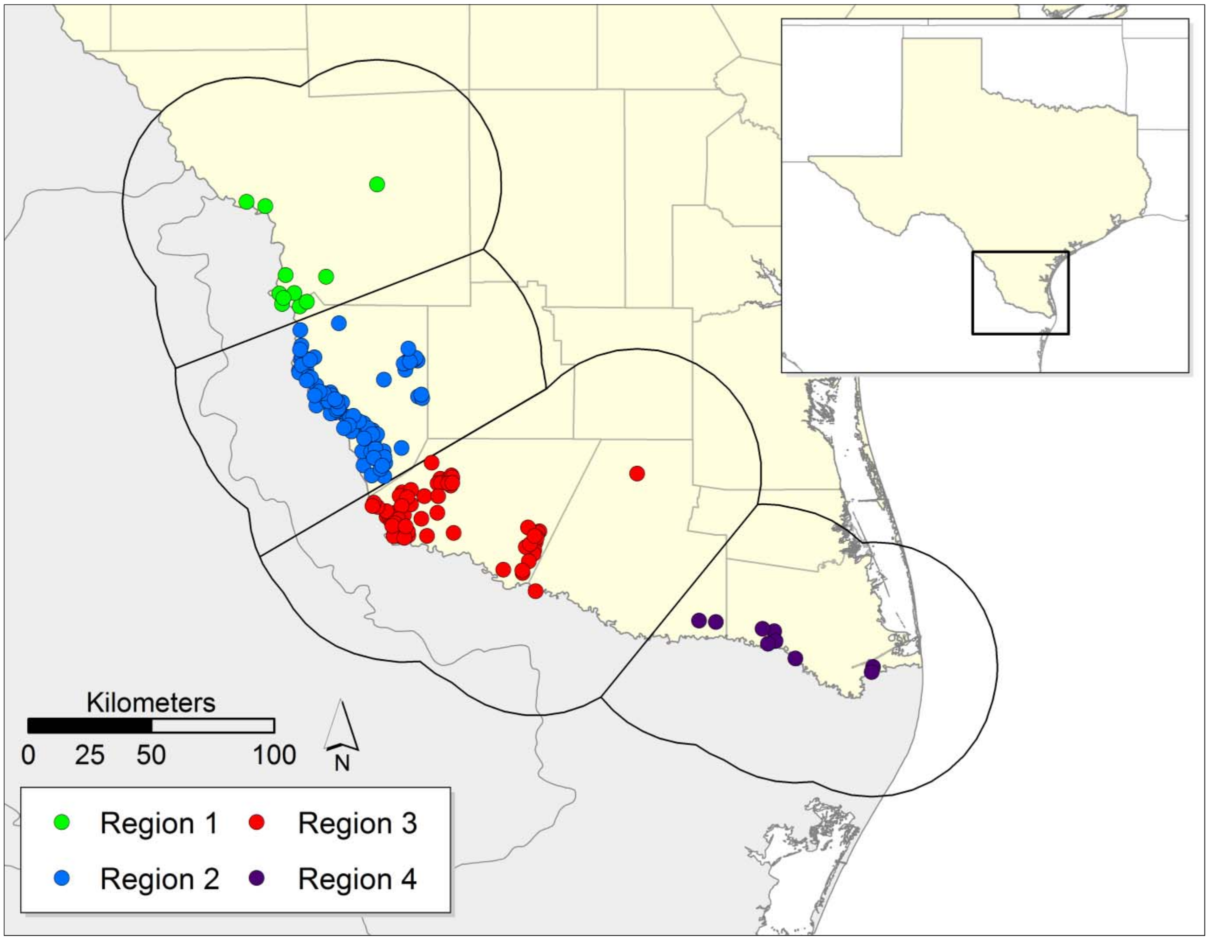

Figure 1. Subdivision of testing regions for *R. microplus* occurrences.

References

1. Elith J, Leathwick JR: **Species distribution models: ecological explanation and prediction across space and time.** *Annual Review of Ecology, Evolution, and Systematics* 2009, **40:**677-697.
2. Barry S, Elith J: **Error and uncertainty in habitat models.** *Journal of Applied Ecology* 2006, **43:**413-423.
3. Peterson A, Papeş M, Eaton M: **Transferability and model evaluation in ecological niche modeling: a comparison of GARP and Maxent.** *Ecography* 2007, **30:**550-560.
4. Ortega-Huerta MA, Peterson AT: **Modeling ecological niches and predicting geographic distributions: a test of six presence-only methods.** *Revista Mexicana de la Biodiversidad* 2008, **79:**205-216.
5. Soberon J, Nakamura M: **Niches and distributional areas: concepts, methods, and assumptions.** *Proceedings of the National Academy of Sciences* 2009, **106:**19644-19650.
6. Elith J, Graham CH, Anderson RP, Dudik M, Ferrier S, Guisan A, Hijmans RJ, Huettman F, Leathwick JR, Lehmann A, et al: **Novel methods improve prediction of species' distributions from occurrence data.** *Ecography* 2006, **29:**129-151.
7. Lobo JM, Jiménez-Valverde A, Hortal J: **The uncertain nature of absences and their importance in species distribution modelling.** *Ecography* 2010, **33:**103-114.
8. Lobo JM, Jiménez-Valverde A, Real R: **AUC: a misleading measure of the performance of predictive distribution models.** *Global Ecology and Biogeography* 2008, **17:**145-151.
9. Elith J, Phillips SJ, Hastie T, Dudík M, Chee YE, Yates CJ: **A statistical explanation of MaxEnt for ecologists.** *Diversity and Distributions* 2011, **17:**43-57.
10. Barve N, Barve V, Jiménez-Valverde A, Lira-Noriega A, Maher SP, Peterson AT, Soberon J, Villalobos F: **The crucial role of the accessible area in ecological niche modeling and species distribution modeling.** *Ecological Modelling* 2011, **222:**1810-1819.
11. Phillips SJ, Dud M, Schapire RE: **A maximum entropy approach to species distribution modeling.** In *Proceedings of the twenty-first international conference on Machine learning*. Banff, Alberta, Canada: ACM; 2004.
12. Stockman A, Beamer D, Bond J: **An evaluation of a GARP model as an approach to predicting the spatial distribution of non-vagile invertebrate species.** *Diversity and Distributions* 2006, **12:**81-89.
13. Stockwell D, Peters D: **The GARP modelling system: problems and solutions to automated spatial prediction.** *International Journal of Geographical Information Science* 1999, **13:**143-158.
14. Giles J, Peterson AT, Almeida A: **Ecology and geography of plague transmission areas in northeastern Brazil.** *PLoS Neglected Tropical Diseases* 2011, **5**.
15. Anderson RP, Lew D, Peterson AT: **Evaluating predictive models of species' distributions: criteria for selecting optimal models.** *Ecological Modelling* 2003, **162:**211-232.
16. Phillips SJ, Dudík M: **Modeling of species distributions with Maxent: new extensions and a comprehensive evaluation.** *Ecography* 2008, **31:**161-175.

17. Davey RB, Cooksey LM, Despins JL: **Survival of larvae of *Boophilus annulatus, Boophilus microplus,* and *Boophilus hybrids* (Acari: Ixodidae) in different temperature and humidity regimes in the laboratory.** *Veterinary Parasitology* 1991, **40:**305-313.
18. Hitchcock LF: **Studies on the non-parasitic stages of the cattle tick, *Boophilus microplus* (Canestrini) (Acari: Ixodidae).** *Australian Journal of Zoology* 1955, **3:**295-311.
19. Pearson RG, Raxworthy CJ, Nakamura M, Peterson AT: **Predicting species distributions from small numbers of occurrence records: a test case using cryptic geckos in Madagascar.** *Journal of Biogeography* 2007, **34:**102-117.
20. Pepe MS: **Receiver operating characteristic methodology.** *Journal of the American Statistical Association* 2000, **95:**308-311.
21. Jiménez-Valverde A: **Insights into the area under the receiver operating characteristic curve (AUC) as a discrimination measure in species distribution modelling.** *Global Ecology and Biogeography* 2011, **21:**498-507.
22. Peterson AT, Papeş M, Soberón J: **Rethinking receiver operating characteristic analysis applications in ecological niche modelling.** *Ecological Modelling* 2008, **213:**63-72.
23. Barve N: **Tool for Partial-ROC.** 1.0 edition. Lawrence, KS: University of Kansas Biodiversity Institute; 2008.
